# Many Mickles Make a Muckle: Evidence That Gender Stereotypes Reemerge Spontaneously Via Cultural Evolution

**DOI:** 10.1177/01461672241254695

**Published:** 2024-06-03

**Authors:** Carolyn J. Dallimore, Kenny Smith, Jacqui Hutchison, Gillian Slessor, Douglas Martin

**Affiliations:** 1 University of Aberdeen, UK; 2 The University of Edinburgh, UK

**Keywords:** stereotypes, gender stereotyping, culture and cognition, social cognition, social bias

## Abstract

We explore whether societal gender stereotypes re-emerge as social information is repeatedly passed from person to person. We examined whether peoples’ memories of personality attributes associated with female and male social targets became increasingly consistent with societal gender stereotypes as information was passed down social transmission chains. After passing through the memories of just four generations of participants, our initially gender-balanced micro-societies became rife with traditional gender stereotypes. While we found some evidence of the re-emergence of gender stereotypes in Experiment 1, we found the effects were stronger when targets appeared in a feminine-stereotyped occupational context (Experiment 2), and a masculine-stereotyped occupational context (Experiment 3); conversely, the re-emergence of gender stereotypes was attenuated when targets appeared in a single gender context (Experiment 4). The current findings demonstrate that gender schematic memory bias, if widely shared, might cause gender stereotypes to be maintained through cultural evolution.

*“There is an old Scotch (sic) adage. . .which none in the whole catalogue of them is more true or more worthy of being held in remembrance, viz*, “that many mickles make a muckle,” *indicating that however trifling a thing may be in itself when it stands alone, when they come to be multiplied, they mount high. . ..”*

George Washington in a letter to James Germain, 1794 (as cited in *Writings of George Washington from the Original Manuscript*, 1931, p. 390)

## Introduction

The current research aimed to establish whether societal gender stereotypes persist because stereotype-consistent memory bias incrementally shapes how social information evolves as it is repeatedly passed from person to person. It is striking that the content of gender stereotypes has remained relatively stable over time despite huge societal change, apparently pervasive changes in people’s attitudes, and extensive interventions to challenge stereotype content (Donnelly & Twenge, 2017; Eagly et al., 2020; Haines et al., 2016; Lueptow et al., 2001). Women are still stereotypically associated with *femininity, communion*, and *warmth*, while men are still stereotypically associated with *masculinity, agency*, and *competence* (Abele & Hauke, 2020). When people endorse these gender stereotypes, it can lead to overt prejudice and discrimination (e.g., Bem, 1981; Cundiff & Vescio, 2016; Devine, 1989); however, even if people do not endorse stereotypes, knowledge of their content can still lead to bias in thoughts and behavior (e.g., Begeny et al., 2020; Bem, 1981; Greenwald & Banaji, 1995). While there is abundant evidence that stereotypes represent a fundamental source of social and cognitive bias, there remains a substantial gap in understanding how and why stereotype content remains so impervious to societal change (Ellemers, 2018). Addressing this gap, the current research investigated whether stereotype-consistent memory bias shapes the evolution of socially transmitted information, resulting in the spontaneous re-emergence of societal gender stereotypes that were initially absent from the social environment.

**Figure 1.**
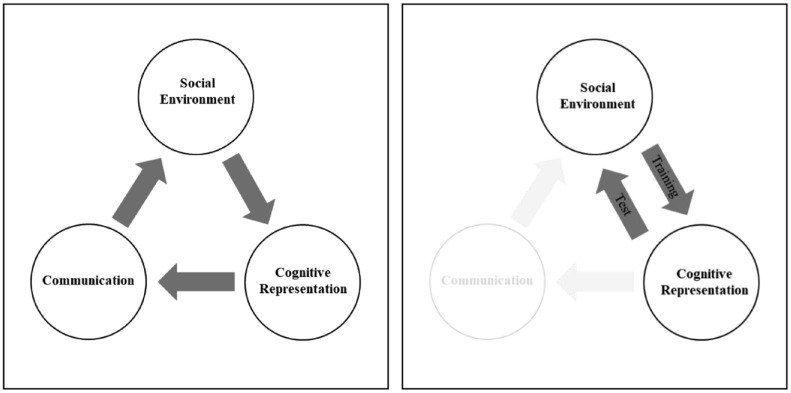
Schematic Depictions of the Proposed Cycle of Cultural Evolution (Left Panel; Adapted From Martin et al., 2017) and the Social Transmission Design of the Current Research (Right Panel).

The central thesis of the current research is that even subtle cognitive bias can accumulate to exert substantial cumulative influence. The cumulative effects of small biases are cornerstones of many scientific disciplines; for example, evolutionary biology (e.g., where small selective differences drive the evolution of species), geology (e.g., where tiny incremental changes shape the Earth’s topography), and climatology (e.g., where the decisions of individual humans collectively impact the global climate) provide notable examples of biases that are barely perceptible at the micro-level but that can have fundamental influence at the macro-level. As a discipline, psychology has been slower to embrace the potential macro-level societal effects that can occur as the cumulative consequence of micro-level biases in individual people (although see Bartlett, 1932; Mesoudi, 2011). This seems like a significant oversight. Human society is a consequence of a continuous cycle of cultural evolution^1^—people are exposed to information from their social environment, they represent this information cognitively, and they can communicate this information to other people, thereby creating a novel social environment from which others can learn (see Figure 1, left panel). We suggest every stage of this cycle is subject to bias, and when these biases are widely shared, they can exert directional pressure on the way information evolves—in the current context, bias toward stereotype-consistent cognitive representation exerts directional pressure that results in the re-emergence of gender stereotypes that were initially absent from the social environment.

The gender stereotypes of feminine women and masculine men seem to have changed surprisingly little in the past 70 years (Donnelly & Twenge, 2017; Eagly et al., 2020; Haines et al., 2016; Lueptow et al., 2001). Evidence for the persistence of traditional gender stereotypes can be seen in explicit self-report measures of stereotype knowledge (Eagly et al., 2020; Haines et al., 2016) and in evidence from implicit reaction time measures (e.g., Nosek et al., 2009). Research using the Bem Sex Role Inventory (Bem, 1981)—a measure of people’s self-identification with gender stereotypes—also reveals that women are more likely to identify with stereotypically feminine attributes and men are more likely to identify with stereotypically masculine attributes; indeed, the difference between women and men’s gender stereotype self-identification has remained relatively consistent for more than 40 years (Donnelly & Twenge, 2017; note that women’s self-identification with masculine trait scores has increased over time, but the difference between genders has remained constant). Given that the last 70 years have seen unprecedented narrowing of gender disparities in many areas of life, such as the number of women and men in both the workplace and higher education, and apparent changes in people’s attitudes toward sex and gender roles, why is it that the content of gender stereotypes remains so stubbornly impervious to change?

One reason why the content of gender stereotypes might be so pervasive is because of the biasing effect they have on cognitive representation. According to gender schema theory, knowledge of gender stereotypes influences the way people cognitively assimilate social information (Bem, 1981). Thus, irrespective of whether a person endorses stereotype content, it influences the way they attend to, store in memory, and recall social information (Fyock & Stangor, 1994; Macrae & Bodenhausen, 2000; Rojahn & Pettigrew, 1992; Sebastián-Tirado et al., 2023; Stangor & McMillan, 1992). It has been shown repeatedly that people are better at remembering information that is consistent with their knowledge of stereotypes relative to information that is inconsistent with these stereotypes (for meta-analyses see Fyock & Stangor, 1994; Stangor & McMillan, 1992; although for an alternative interpretation see Rojahn & Pettigrew, 1992). Similarly, there is evidence that people experience stereotype-consistent memory intrusions that lead them to falsely believe they have encountered stereotype-consistent information that was never present (Bellezza & Bower, 1981; Lenton et al., 2001; Sebastián-Tirado et al., 2023). Such stereotype-consistent memory biases have the potential to influence the way individual people cognitively (mis)represent their own experience of social reality (Bem, 1981).

Any bias in memory for information consistent with gender schema (Bem, 1981)—whether it is a bias in favor of correctly recalling stereotype-consistent information (Fyock & Stangor, 1994) or a bias toward making stereotype-consistent memory intrusions (Bellezza & Bower, 1981; Lenton et al., 2001; Sebastián-Tirado et al., 2023)—need not be large to exert substantial influence on our collective culture and society. Even if the bias individual people exhibit is very small this can exert huge societal influence when many people possess the same or similar biases and when information is repeatedly socially transmitted (see Mesoudi, 2011). The cumulative effect of widely shared biases—often called *cultural evolution*—is thought to be fundamental to the development of many aspects of human culture (Boyd & Richerson, 1988; Kirby et al., 2008; Sperber, 1996). If enhanced memory for stereotype-consistent information is a widely shared cognitive bias, then even if this bias is subtle, even if it is not universally present in all people, and even if it is not always evident, it is plausible that the effects of this bias could accumulate to maintain societal stereotypes as information is repeatedly communicated from person to person.

Evidence from communication research suggests social information evolves to become increasingly stereotype-consistent when it is repeatedly communicated (e.g., Kashima, 2000). In dyadic conversation, people spend more time discussing stereotype-consistent information, expressing agreement with stereotype-consistent statements, and focusing questions and discussion on stereotype-consistent information (see Ruscher, 1998). This is partly because people tailor the content of their communications based on their perceptions of an audience’s knowledge and expectations—a phenomenon often referred to as “audience tuning” (Echterhoff et al., 2005). These stereotype-consistent biases in communication can accumulate, with evidence that when stories are repeatedly passed from person to person, they become increasingly consistent with existing gender stereotypes (e.g., Clark & Kashima, 2007; Kashima, 2000; Lyons & Kashima, 2003). The prevailing thought is that people possess *social biases* and *communicative biases* that make them more likely to communicate stereotype-consistent information (for a review see Kashima et al., 2007). However, because all communication requires recalling information from memory, if people possess *cognitive biases* that mean they are more likely to recall stereotype-consistent information (Fyock & Stangor, 1994), this might increase the likelihood of stereotype-consistent communication without the need for social and communicative biases. Thus, even in the absence of social and communicative pressure, stereotype-consistent memory bias might cause information to become increasingly stereotype-consistent as it is repeatedly passed from person to person. A central aim of the current research was therefore to establish whether, in the absence of communication bias, social information evolves to become increasingly stereotype-consistent due to memory bias in cognitive representation alone.

Recent research suggests that stereotype content might spontaneously form and change via cultural evolution without the need for social or communicative pressure (Hutchison et al., 2018; Hutchison & Martin, 2015; Martin et al., 2014, 2017). Martin et al. (2014) examined the formation and evolution of novel stereotypes using a transmission chain method, where information is repeatedly passed from person to person in a process akin to the children’s game often called “Chinese Whispers” in the United Kingdom or “Telephone” in the United States. Participants attempted to learn attributes that had been randomly associated with “alien beings” that were individually unique but that also shared category membership (e.g., some aliens were the same shape, some were the same color). Whichever attributes one participant recalled as being associated with a particular alien were used as the basis of the training materials for the next participant in the chain of transmission. Crucially, there was no social or communicative pressure, as participants were not aware that they were part of a transmission chain. An initially random set of attributes that was difficult to remember became increasingly simplified and learnable as it passed from person to person. Even small tendencies toward structure evident in the attribute assignments made by one participant were detected and amplified by the next. Over multiple such “generations” of transmission, a systematic stereotype-like relationship developed, with categorical features becoming so strongly associated with specific attributes that they could be used to accurately infer information about aliens that had never been seen before (e.g., by the end of one chain all green aliens were agreed to be *arrogant* and *pushy*, while red aliens were thought to be *shy*). These findings support the possibility that the formation and evolution of stereotypes is driven by shared cognitive biases, the effects of which accrue as information is repeatedly transmitted between people.

## Aims and Hypotheses

The overarching aim of the current research was to establish whether existing stereotypes re-emerge and persist because stereotype-consistent memory bias shapes how social information evolves as it is repeatedly passed from person to person. We know from previous research that information becomes increasingly stereotype-consistent when people are asked to communicate their knowledge to others (Clark & Kashima, 2007; Kashima, 2000; Kashima et al., 2007; Lyons & Kashima, 2003); however, it is not clear whether these effects are reliant on social and communicative biases or whether similar effects might be driven by a stereotype-consistent memory bias (Bem, 1981; Fyock & Stangor, 1994; Lenton et al., 2001). We also know from previous research that a bias toward making within-category memory errors can drive the spontaneous formation of novel stereotypes (Hutchison et al., 2018; Martin et al., 2014). Because micro-level gender schema are generally acknowledged to be widely shared across the population (possibly universally shared; Bem, 1981), they are an example of the kind of widely shared cognitive bias that is likely to influence macro-level cultural evolution. Is it possible, then, that a memory bias for stereotype-consistent information might lead to the re-emergence of existing stereotypes as social information is repeatedly socially transmitted?

The current research uses a “social transmission chain” design to study how stereotype driven bias in cognitive representation influences cultural evolution in a lab-based setting. In this design, people are trained on information from a novel social environment and their memory is subsequently used as the training materials for the next person in a chain. This process is repeated to create transmission chains of multiple generations, thereby mimicking how cultural knowledge is passed down through generations. Crucially, in our design, participants are not asked to communicate information to other people but rather are only asked to recall it from memory (indeed they are not even aware that they are part of a transmission chain). This allows us to investigate whether cognitive bias associated with knowledge of stereotypes alone can cause stereotypes to spontaneously re-emerge in an environment that is initially free from bias and where there is no bias from communicative pressure (see Figure 1, right panel).

We investigated whether gender stereotype-consistent memory bias shapes the cultural evolution of socially transmitted information, resulting in the spontaneous re-emergence of societal gender stereotypes that were initially absent from the social environment. Adapting the transmission chain method previously used to examine how novel stereotypes spontaneously form and evolve (Martin et al., 2014), across four experiments we examined whether people’s memory for the personality attributes associated with female and male social targets became increasingly gender stereotype-consistent as information passed down transmission chains of four generations of participants. If gender schema drives a stereotype-consistent memory bias (Bem, 1981; Fyock & Stangor, 1994; Lenton et al., 2001), information should evolve to become increasingly stereotype-consistent as it is repeatedly passed from person to person.

## Experiment 1

The aim of Experiment 1 was to examine whether gender stereotypes spontaneously re-emerge through a process of cultural evolution. Specifically, we wanted to examine whether repeatedly passing peoples’ memories for the attributes associated with social targets down transmission chains would result in information becoming increasingly consistent with societal gender stereotypes. Participants arrived at the lab and were assigned to a novel “team” and were told that their tasks during the experiment would be to learn who was in each team,^2^ learn attributes associated with each person, and finally, try to remember which attributes went with which person. Participants were asked to learn personality attributes associated with sixteen social targets (8 female targets and 8 male targets). We initialized each chain by assigning to each target two stereotypically feminine attributes (e.g., *caring*), two stereotypically masculine attributes (e.g., *arrogant*), and two gender stereotype-neutral attributes (e.g., *secretive*); we refer to this initial set of target-attribute assignments as *Generation 0*. Importantly, female and male targets were assigned identical attributes at Generation 0 across the chains. During an initial training phase, the first participant in a chain (Generation 1) attempted to learn which attributes went with which target person. During a subsequent recall phase, the participant was shown each target person again and asked to identify their attributes. The attribute-to-target pairings each person produced during the recall phase were used as the training materials for the next participant in the chain (Generation 2). The process of using the test responses from one generation as the training materials for the next was repeated three times per chain to create social transmission chains of four generations (i.e., Generation 1-4).

Because people possess gender schema that causes them to show a bias for remembering stereotype-consistent information (Bem, 1981; Fyock & Stangor, 1994; Stangor & McMillan, 1992), we hypothesized that passing initially gender-balanced social information through the memories of just four generations of participants would result in the spontaneous re-emergence of societal gender stereotypes. We expected the accumulation of stereotype-consistent information to be indexed in target-attribute pairings in two ways: first, we hypothesized that by the end of the chains the absolute frequency of stereotype-consistent target-attribute pairings would be greater than at the start of the chains (i.e., relative to the start of the chains, by Generation 4 female targets would be associated with a higher frequency of feminine attributes and male targets would be associated with a higher frequency of masculine attributes). Second, we hypothesized that by the end of the chains there would be a higher frequency of stereotype-consistent target-attribute pairings than stereotype-inconsistent target-attribute pairings (i.e., by the end of the chains feminine attributes would be paired relatively more frequently with female targets than male targets, and masculine attributes would be paired relatively more frequently with male targets than female targets).

## Method

The experiments were designed as a sub-project of a successful grant application (ES/N019121/1), in 2016, before it was commonplace for research designs to be preregistered; consequently, no studies reported in this manuscript were preregistered. We report all manipulations, measures, and exclusions in these studies (although see footnote 1). All data, variable codebook, analysis code, and verbatim wording of participant instructions, have been made publicly available at the *Open Science Foundation* and can be accessed at https://osf.io/nf2pj/?view_only=4a719fd5eaae4b158385c070e2f3f181.

## Participants

The participants were 64 students (52 female and 12 male) who were granted course credit in exchange for taking part in the experiment. The participants were assigned to sequential generations in 1 of 16 active chains and were given full instructions both verbally and onscreen. The sample size was based on previous research (Martin et al., 2014) which indicated consistent stereotype formation after four generations of transmission. *A priori* power analyses conducted using GPower (Faul et al., 2009) show sufficient power (over .90 on every measure) to detect the effect sizes observed in Martin et al. (2014) with a minimum of 13 chains of four generations each.

## Materials

### Social Targets

Each social target was represented by 1 of 16 color front-facing digital headshot images of unfamiliar people (8 female and 8 male) selected from the Chicago Face Database (Ma et al., 2015), cropped to a standard size of 200 × 240 pixels (1,280 × 1,024 screen resolution). To minimize bias associated with other social categories, all target identities were White younger adults, which reflected the racial identity and age of our participant population. We used the norms available from the Chicago Face Database to minimize between-sex differences in physical and perceived psychological characteristics (i.e., attractiveness, trustworthiness, and distinctiveness, age; see Supplementary Tables A-B).

### Attributes

The attributes used to describe each social target were drawn from a total pool of 48 attributes that could be used to describe a human. The chosen attributes were based on gender-stereotyped and gender-neutral items that appear in the Bem Sex Role Inventory or associated synonyms (Bem, 1981);^3^ there were 16 stereotypically feminine attributes (e.g., *caring*), 16 stereotypically masculine attributes (e.g., *arrogant*), and 16 gender stereotype-neutral attributes (e.g., *secretive*). Six attributes were used to describe each target.

## Procedure

*Generating the initial system (Generation 0)*: We created Generation 0 by assigning six attributes to each target in a pseudo-random way that minimized the categorical structure associated with the target sex. Each social target was assigned two stereotypically feminine attributes (e.g., *caring*), two stereotypically masculine attributes (e.g., *arrogant*), and two relatively gender stereotype-neutral attributes (e.g., *secretive*). Importantly, female and male targets were each collectively assigned identical attributes at Generation 0 across the chains. The specific assignment of attributes to targets was counterbalanced across chains.

*Stage 1. Minimal group induction*: Participants were assigned to one of two teams within the experiment and were given 2 min to study two office layouts, which included images of each team member and where they supposedly sat. Participants were then tested on their knowledge of the members of both teams by being asked to remember where each person sat and via a simple face recognition test.
*Stage 2. Attribute training*: Participants were told that they would now be presented with personality attributes that described each of their colleagues and that their task was to form an impression of each person and to remember their attributes. Each trial lasted 20-s and comprised the presentation of a face image and six personality attributes that described the person. To aid learning, participants viewed each target and their associated information three times (i.e., 48 trials in total). The order of target presentation was randomized across trials.
*Stage 3. Attribute memory test*: Participants were asked to try to remember which attributes went with which targets in the attribute training stage (stage 2). Each trial comprised the presentation of a single target face below a list of the entire pool of 48 possible attributes. Participants completed 16 trials, one per target person, with the trial order randomized. Each target remained onscreen until six attributes had been chosen, with participants encouraged to make their best guess if they were unsure about which attributes to choose.

*Social transmission of information (Generations 1-4)*: Following each participant’s completion of the experiment, the attributes they assigned during the memory test stage (Stage 3) were transmitted as the materials for the attribute training stage of the next participant (Stage 2). The process of transmitting the memory test responses from one participant as the impression formation materials for the next was repeated to create continuous transmission chains of four generations.

## Dependent Measures and Analysis Strategy

*Frequency of stereotype-consistent and stereotype-inconsistent target-attribute pairings over time*: To establish whether information became increasingly stereotype-consistent as it passed down the chains we analyzed the mean proportion target-attribute frequencies with 2 (Generation: G1 vs. G4) × 2 (Target Sex: female targets vs. male targets) × 2 (Attribute Type: stereotype-consistent vs. stereotype-inconsistent)^4^ repeated measures analysis of variance (ANOVA). If information became increasingly stereotype-consistent over time we expected to find a significant Generation × Attribute type interaction driven by a significant increase in stereotype-consistent target-attribute pairings but not stereotype-inconsistent target-attribute pairings.

## Experiment 1 Results

*Frequency of stereotype-consistent and stereotype-inconsistent target-attribute pairings over time*: The only significant effect to emerge from the analysis was the main effect of Generation (*F*[1, 15] = 22.41, *p* < .001; η^2^ = .599; see Supplementary Table 1 for all cell values]). However, this effect was included in a non-significant trend toward the predicted interaction of Generation × Attribute Type [*F*(1, 15) = 3.99, *p* = .064; η^2^ = .210], which is explored in more detail below. There was no evidence of any other significant main effects or interactions (all *F*s < .174, all *p*s > .120).

**Figure 2.**
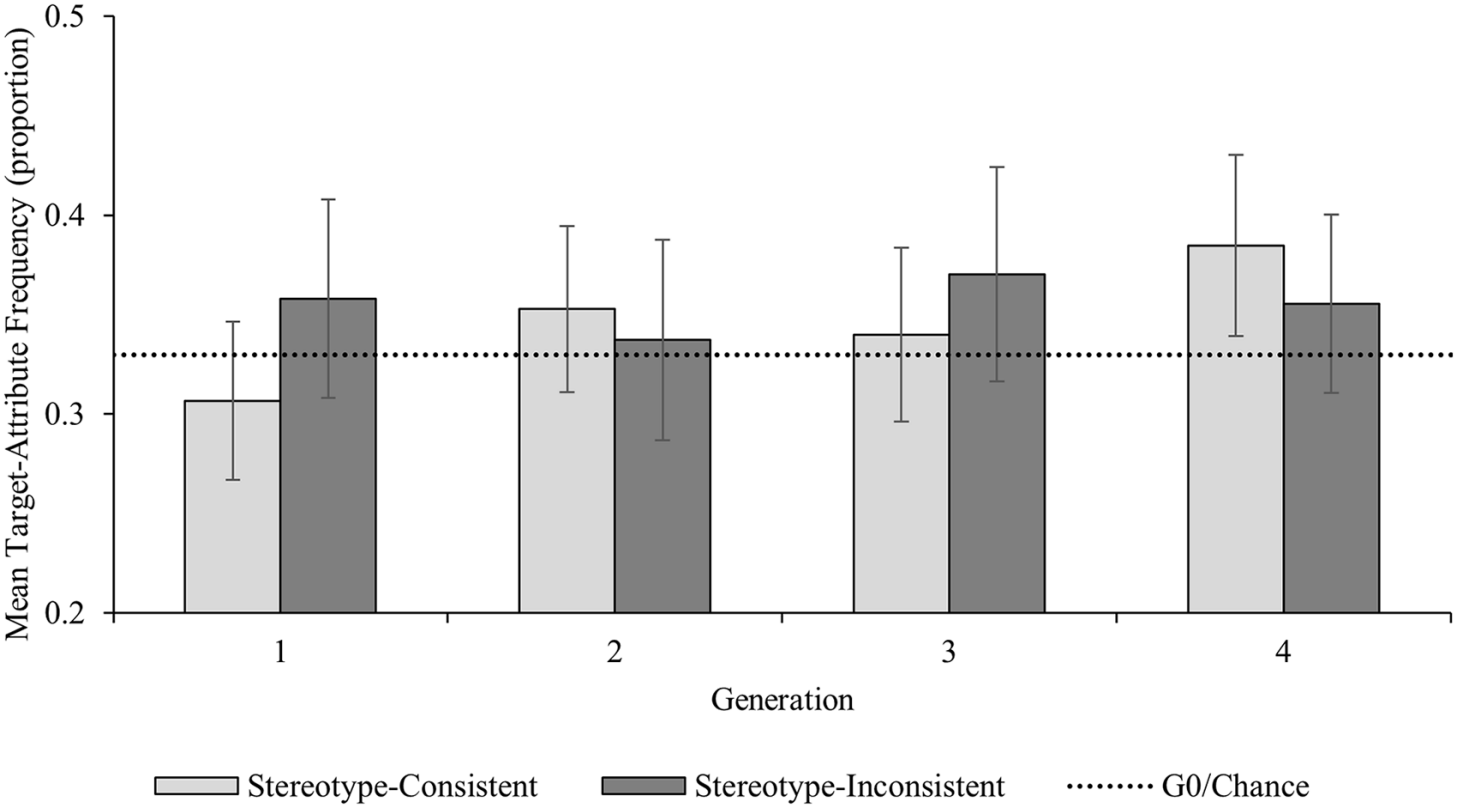
Experiment 1 Frequency of Target-Attribute Pairings by Generation and Attribute Type. *Note*. Error bars represent 95% confidence intervals.

Given its central importance to the current research, we further explored the non-significant trend toward the predicted Generation × Attribute Type interaction; we did this by examining the pairwise comparisons for stereotype-consistent and stereotype-inconsistent target-attribute pairings both within and between each Generation (Figure 2).^5^ As predicted, stereotype-consistent target-attribute pairings increased significantly between G1 (*M prop* = .31; 95% confidence interval [CI]: [.27, .35]) and G4 (*M prop* = .39; 95% CI [.34, .43]; *M diff* = .08 (95% CI [.03, .13]; *p* = .003; *d* = .88, 95% CI [.29, 1.45]); there was no change in the proportion of stereotype-inconsistent target-attribute pairings between G1 (*M prop* = .36; 95% CI [.31, .41]) and G4 (*M prop* = .36; 95% CI [.31, .40]; *M diff* = .003 (95% CI [−.04, .04]; *p* = .904; *d* = .03, 95% CI [-.46, .52]). There was no difference in the frequency of stereotype-consistent and stereotype-inconsistent attributes at G1 (*M diff* = .05; 95% CI [−.03, .14]; *p* = .209; *d* = .33, 95% CI [−.18, .83]); contrary to our predictions, there was also no difference in the frequency of stereotype-consistent and stereotype-inconsistent attributes at G4 (*M diff* = .03; 95% CI [−.05, .11]; *p* = .463; *d* = .19, 95% CI [−.31, .68]).

## Discussion—Experiment 1

The aim of Experiment 1 was to examine whether gender stereotypes spontaneously re-emerge through a process of cultural evolution—we found only limited evidence that this was the case. As predicted, there was evidence that stereotype-consistent information accumulated as it passed down the chains, with significantly more stereotype-consistent target attributes at the end of the chains than at the start. However, contrary to our prediction, there was no evidence that by the end of the chains there would be more stereotype-consistent target-attribute pairings than stereotype-inconsistent target-attribute pairings.

While we found some evidence that information evolved to become increasingly consistent with gender stereotypes as it passed down the chains, there are several reasons why this might not have culminated in the spontaneous re-emergence of gender stereotypes by the end of the chains. Directional cultural evolution of information depends on the strength of biases of individual learners, how many people share these biases, and how often information is communicated (Hutchison et al., 2018; Martin et al., 2017; Mesoudi, 2011). It is likely that the current experimental context resulted in an individual memory bias toward stereotype-consistent information that was smaller than we anticipated (Fyock & Stangor, 1994; Lenton et al., 2001). Given the frequency of stereotype-consistent target-attribute pairings was on an increasing trend, with substantial increases in stereotype-consistent pairings between Generation 3 and Generation 4, it is likely this level of bias might result in stereotype re-emergence with more generations of participants per chain. However, rather than examining the cumulative effects of weak bias over a greater number of generations, an alternative approach is to examine whether stronger bias might result in the re-emergence of existing stereotypes over the same number of generations—we explored whether a stronger gender stereotype-consistent memory bias would lead to the re-emergence of gender stereotypes in Experiment 2.

## Experiment 2

The aim of Experiment 2 was to induce a stronger stereotype-consistent memory bias, by introducing a novel gendered occupational context, to examine whether gender stereotypes spontaneously re-emerge through a process of cultural evolution. Gender-based occupational stereotypes are very prevalent, with jobs requiring communal skills more likely to be perceived as feminine and undertaken by women (e.g., nurses, secretaries, and elementary school teachers), and jobs requiring agentic skills more likely to be perceived as masculine and undertaken by men (e.g., engineers, computer programmers, and surgeons; Atli, 2017; Matheus & Quinn, 2017; Treleaven, 2015; White & White, 2006). Recent research suggests gender schema of occupations can lead to stereotype-consistent memory bias, with people more likely to correctly and falsely recall information that is consistent with occupational gender stereotypes (Sebastián-Tirado et al., 2023). It is therefore possible the presence of gendered occupational context might lead to a stronger gender stereotype-consistent memory bias, which in turn leads to the re-emergence of gender stereotypes over time. Thus, in Experiment 2, we examined whether a feminine-stereotyped social environment influenced the maintenance of gender stereotypes, by describing targets as belonging to one of two occupational teams with novel feminine-stereotyped names (i.e., *community outreach team* or *social resources team*). We chose to use novel occupational titles, which were aligned with gender stereotypes but which were not themselves associated with strong occupational stereotypes. This is because we were concerned that using strongly gendered real-world roles (e.g., nurse and surgeon) would result in the re-emergence of stereotypes associated with those occupations/settings rather than gender per se. Our predictions were the same as those in Experiment 1.

## Method

### Participants

The participants were 64 students (60 female and 4 male) from the University of Aberdeen, who were granted course credit in exchange for taking part in the experiment.

### Materials

The materials used were identical to those of Experiment 1, except that references to “your team” and “the other team” were replaced with “community outreach team” and “social resources team” respectively; pilot testing indicated that these fictitious job titles were strongly associated with feminine-stereotyped attributes (see Supplementary Tables E-F).

### Procedure

The procedure was the same as that of Experiment 1.

### Dependent Measures and Analyses

The dependent measures and analyses were identical to those used in Experiment 1.

## Experiment 2 Results

*Frequency of stereotype-consistent and stereotype-inconsistent target-attribute pairings over time*: We analyzed the data for the mean proportional frequency with which female and male targets were paired with feminine and masculine stereotyped attributes at the start and end of the chains using a 2 (Generation: G1 vs. G4) × 2 (Target Sex: female targets vs. male targets) × 2 (Attribute Type: stereotype-consistent vs. stereotype-inconsistent) repeated measures ANOVA (see Supplementary Table 2 for all cell values). The analysis revealed a main effect of Attribute Type [*F*(1, 15) = 7.35, *p* = .016; η^2^ = .329], but this was subsumed by the predicted interaction of Generation × Attribute Type [*F*(1, 15) = 5.20, *p* = .038; η^2^ = .257], which is explored in more detail below. There was no evidence of any other significant main effects or interactions (all *F*s < .467, all *p*s > .504).

**Figure 3.**
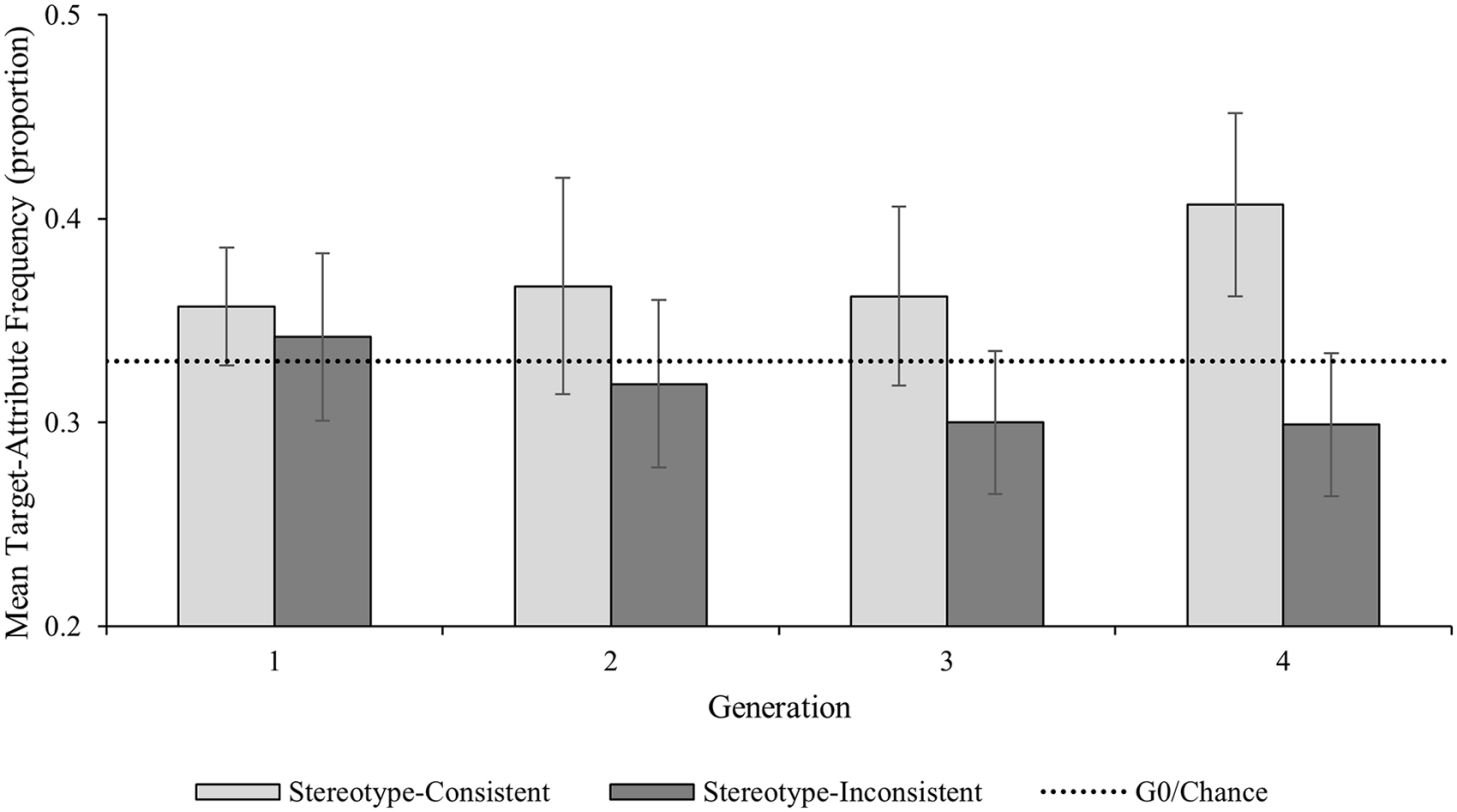
Expt. 2 Frequency of Target-Attribute Pairings by Generation and Attribute Type. *Note.* Error bars represent 95% confidence intervals.

We explored the predicted Generation × Attribute Type interaction by examining the pairwise comparisons for stereotype-consistent and stereotype-inconsistent target-attribute pairings both within and between Generation 1 and Generation 4 (Figure 3). As predicted, stereotype-consistent target-attribute pairings increased significantly between G1 (*M prop* = .36; 95% CI [.33, .39]) and G4 (*M prop* = .41; 95% CI [.36, .45]; *M diff* = .05 (95% CI [.01, .09]; *p* = .018; *d* = .66, 95% CI [.11, 1.19]); there was no change in the proportion of stereotype-inconsistent target-attribute pairings between G1 (*M prop* = .34; 95% CI [.30, .38]) and G4 (*M prop* = .29; 95% CI [.26, .34]; *M diff* = .04 (95% CI [−.01, .10]; *p* = .109; *d* = .43, 95% CI [−.09, .93]). There was no difference in the proportion of stereotype-consistent attributes and stereotype-inconsistent attributes used at G1 (*M diff* = .02; 95% CI [−.05, .08]; *p* = .608; *d* = .13, 95% CI [−.36, .62]); however, as predicted there was a significantly higher proportion of stereotype-consistent than stereotype-inconsistent target-attribute pairings at G4 (*M diff* = .11; 95% CI [.04, .17]; *p* = .003; *d* = .87, 95% CI [.28, 1.44]).

## Discussion—Experiment 2

The aim of Experiment 2 was to induce a stronger stereotype-consistent memory bias, by introducing a gendered occupational context, to examine whether gender stereotypes spontaneously re-emerge through a process of cultural evolution—we found evidence that this was the case. As predicted, and as in Experiment 1, there was evidence that stereotype-consistent information accumulated as it passed down the chains, with significantly more stereotype-consistent target attributes at the end of the chains than at the start. However, unlike in Experiment 1, there was also evidence to support our prediction that by the end of the chains there would be more stereotype-consistent target-attribute pairings than stereotype-inconsistent target-attribute pairings. Given that in a stereotypically feminine occupational context female targets became increasingly associated with feminine attributes and male targets became increasingly associated with masculine attributes, one would expect the same pattern in a masculine context, which provides an opportunity to test the replicability of this finding. We explored this possibility in Experiment 3.

## Experiment 3

The aim of Experiment 3 was to examine whether gender stereotypes spontaneously re-emerge through a process of cultural evolution in the context of a masculine occupational context. As was the case in Expt. 2, we created a gendered social context by describing targets as belonging to one of two novel teams, in this case with stereotypically masculine sounding names (i.e., *mobility analyst team* or *product factors team*). Our predictions were the same as those for Experiment 1 and Experiment 2.

## Method

### Participants

The participants were 64 undergraduate students (32 male and 32 female)^6^ from the University of Aberdeen who were granted course credit in exchange for taking part in the experiment.

### Materials

The materials used were identical to those of Experiment 2, except references to the relatively feminine team names were replaced with the masculine team names of “mobility analysts” and “product factors”; pilot testing indicated that these fictitious job titles were strongly associated with masculine stereotyped attributes (see Supplementary Tables E-F).

### Procedure

The procedure was the same as that of Experiment 2.

### Dependent Measures and Analyses

The dependent measures and analyses were identical to those used in Experiments 1 and 2.

## Experiment 3 Results

*Frequency of stereotype-consistent and stereotype-inconsistent target-attribute pairings over time*: We analyzed the data for the mean proportional frequency with which female and male targets were paired with feminine and masculine stereotyped attributes at the start and end of the chains using a 2 (Generation: G1 vs. G4) × 2 (Target Sex: female targets vs. male targets) × 2 (Attribute Type: stereotype-consistent vs. stereotype-inconsistent) repeated measures ANOVA (see Supplementary Table 3 for all cell values). The analysis revealed a main effect of Attribute Type, *F*(1, 15) = 12.30, *p* = .003; η^2^ = .451, but this was subsumed by the predicted interaction of Generation X Attribute Type, *F*(1, 15) = 10.04, *p* = .006; η^2^ = .401], which is explored in more detail below. There were also several unpredicted significant effects and trends, including a significant interaction of Target Sex and Attribute Type [*F*(1, 15) = 7.96, *p* = .013; η^2^ = .347], which is explored in more detail below, a nonsignificant trend toward a main effect of Target Sex [*F*(1, 15) = 4.24, *p* = .057; η^2^ = .220], and a nonsignificant trend toward a three-way interaction [*F*(1, 15) = 3.24, *p* = .092; η^2^ = .178]. There was no evidence of any other significant main effects or interactions (all *F*s < 2.25, all *p*s > .154).

**Figure 4.**
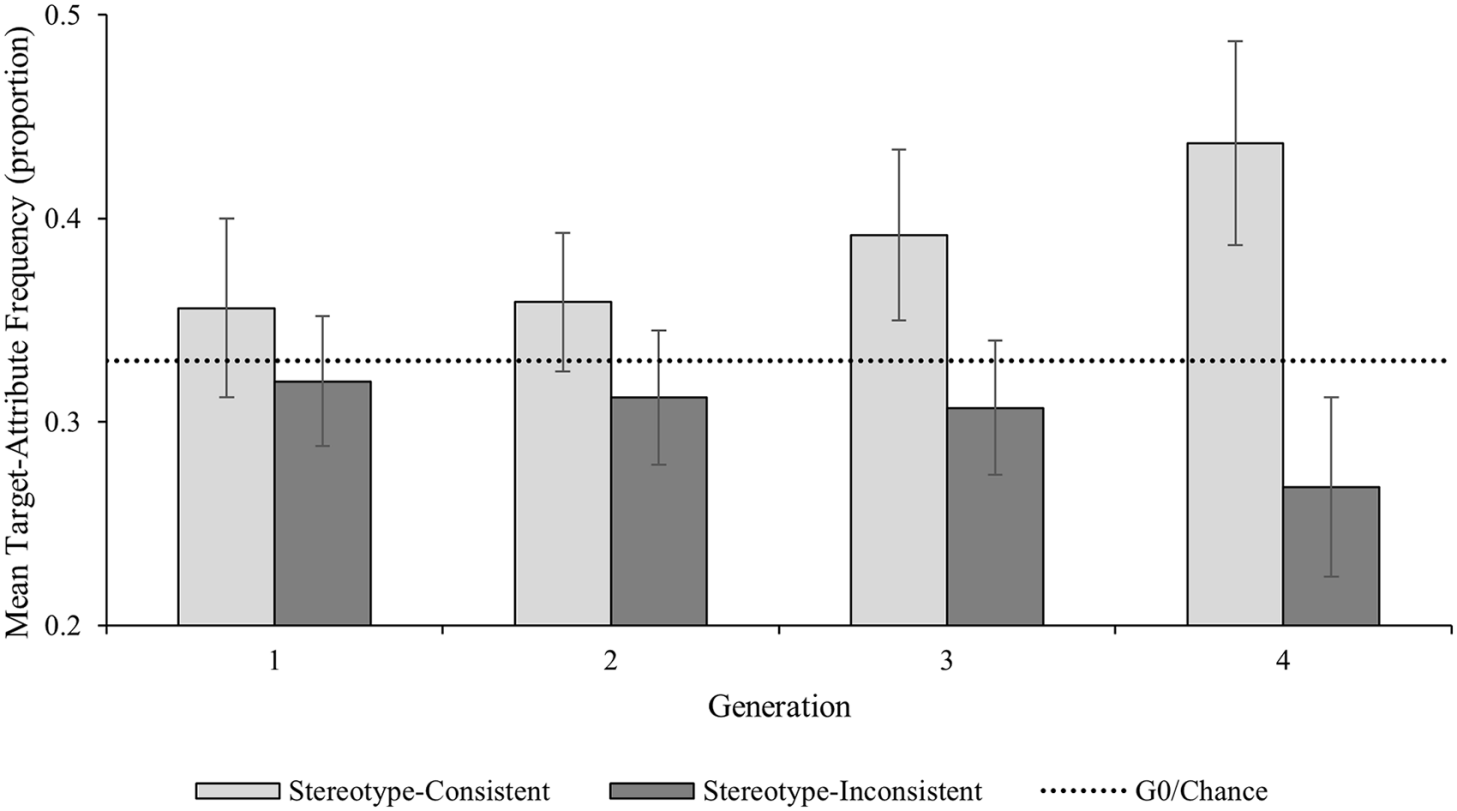
Expt. 3 Frequency of Target-Attribute Pairings by Generation and Attribute Type. *Note*. Error bars represent 95% confidence intervals.

We explored the predicted Generation × Attribute Type interaction by examining the pairwise comparisons for stereotype-consistent and stereotype-inconsistent target-attribute pairings both within and between Generation 1 and Generation 4 (Figure 4). As predicted, stereotype-consistent target-attribute pairings increased significantly between G1 (*M prop* = .36; 95% CI [.31, .40]) and G4 (*M prop* = .44; 95% CI [.39, .49]; *M diff* = .08; 95% CI [.03, .13]; *p* = .005; *d* = .81, 95% CI [.23, 1.37]); conversely, stereotype-inconsistent target-attribute pairings decreased significantly between G1 (*M prop* = .32; 95% CI [.29, .35]) and G4 (*M prop* = .27; 95% CI [.22, .31]; *M diff* = .05 (95% CI [.01, .10]; *p* = .026; *d* = .62, 95% CI [.07, 1.15]). There was no difference in the frequency of stereotype-consistent and stereotype-inconsistent attributes used at G1 (*M diff* = .04; 95% CI [−.03, .11]; *p* = .287; *d* = .28, 95% CI [.23, .77]); however, as predicted, there was a significantly higher frequency of stereotype-consistent than stereotype-inconsistent target-attribute pairings at G4 (*M diff* = .17; 95% CI [.09, .25]; *p* < .001; *d* = 1.09, 95% CI [.45, 1.70]).

Given its potential theoretical interest, we also explored the unexpected Target Sex × Attribute Type interaction, by inspecting the pairwise comparisons for stereotype-consistent and stereotype-inconsistent target-attribute pairings between female and male targets. Stereotype-consistent pairings were significantly more frequent for male targets (*M prop* = .44; 95% CI [.39, .49]) than for female targets (*M prop* = .36; 95% CI [.32, .40]; *M diff* = .08; 95% CI [.03, .14]; *p* = .004; *d* = .86, 95% CI [.27, 1.42]); whereas, there was a nonsignificant trend in the opposite direction for stereotype-inconsistent pairings, which were numerically more frequent for female targets (*M prop* = .32; 95% CI [.28, .36]) than for male targets (*M prop* = .27; 95% CI [.23, .31]; *M diff* = .05; 95% CI [−.01, .01]; *p* = .087; *d* = .46, 95% CI [−.07, .97]).

## Discussion—Experiment 3

The findings from Expt. 3 further support and extend those from Expt. 1 and Expt. 2. There was further evidence that memory bias for stereotype-consistent information accumulated as it passed down the chains. As in both Experiment 1 and Experiment 2, there was evidence that stereotype-consistent information accumulated as it passed down the chains, with significantly more stereotype-consistent target attributes at the end of the chains than at the start. As in Experiment 2, there was also evidence to support our prediction that by the end of the chains, there would be more stereotype-consistent target-attribute pairings than stereotype-inconsistent target-attribute pairings. Unlike in Experiment 1 and Experiment 2, there was also evidence that the size of the stereotype maintenance differed depending on target gender, with male targets becoming more strongly associated with stereotype-consistent attributes than female targets.

## Cross-Experiment Analysis (Experiments 1-3)

We ran a series of cross-experiment analyses to establish whether there was statistical support for the apparent differences between the experimental contexts from Experiments 1 to 3 and to further illuminate the mechanism underlying the effects. The results from Experiments 1 to 3 support the idea that gender stereotypes can re-emerge, through a process of cultural evolution, as social information is repeatedly passed from person to person. However, the pattern of results across the experiments also seems to indicate that the extent of stereotype re-emergence differs depending on the target gender and social context. Based on these emergent patterns in the data, we ran exploratory cross-experiment analyses to test whether there was statistical support for these trends. First, we wanted to determine whether there was greater evidence of stereotype maintenance in more gendered social contexts (Experiments 2 and 3) than in a less gendered social context (Experiments 1). Second, we wanted to establish whether there was greater evidence of stereotype maintenance for male targets than for female targets.

We also used the cross-experiment analyses as an opportunity to explore two additional questions that arose during the peer review process, specifically (a) whether the observed effects were driven by better memory for stereotype-consistent information or a bias toward making stereotype-consistent memory intrusions and (b) whether the size of stereotype-consistent memory bias was the same across the generations or whether it changes as the social environment becomes increasingly stereotype-consistent.

## Results—Cross-Experiment Analysis (Experiments 1 to 3)

We addressed the first three aims of the cross-experiment analysis by analyzing the mean proportional frequency with which female and male targets were correctly and incorrectly paired with feminine and masculine stereotyped attributes at the start and end of the chains using a 3 (Experiment Context: neutral vs. feminine vs. masculine) × 2 (Generation: G1 vs. G4) × 2 (Target Sex: female targets vs. male targets) × 2 (Attribute Type: stereotype-consistent vs. stereotype-inconsistent) × 2 (Accuracy: correct vs. intrusions) mixed ANOVA with Experiment Context as the only between-subjects factor (see Supplementary Table 4 for all cell values). We only describe the effects of central theoretical interest, but a full description of all effects can be found in Supplementary Table 5.

**Figure 5.**
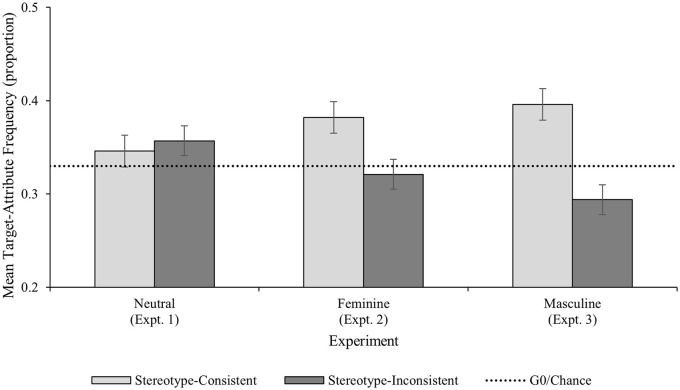
Experiments 1–3 Frequency of Target-Attribute Pairings by Experiment and Attribute Type. *Note.* Error bars represent 95% confidence intervals.

*Is the extent of stereotype maintenance influenced by social context?* Supporting the idea that stereotype maintenance differed in different social contexts, there was evidence of a significant Experiment × Attribute Type interaction, *F*(2, 45) = 4.01, *p* = .025; η^2^ = .151; see Figure 5. Inspection of the pairwise comparisons revealed that the frequency of stereotype-consistent target-attribute pairings was significantly higher in the masculine context (*M prop* = .40; 95% CI [.36, .43]) than in the neutral context (*M prop* = .35, 95% CI [.31, .38]; *M diff* = .05, 95% CI [.003, .10], *p* = .038; *d* = .72, 95% CI [.001, 1.43]) but that there was no difference between the masculine context and the feminine context (*M prop* = .38, 95% CI [.35, .42]; *M diff* = .01, 95% CI [−.03, .06], *p* = .549; *d* = .21, 95% CI [−.48, .91]), or between the feminine context and the neutral context (*M diff* = .04, 95% CI [−.01, .08], *p* = .132; *d* = .57, 95% CI [−.14, 1.28]). The frequency of stereotype-inconsistent target-attribute pairings was significantly lower in the masculine context (*M prop* = .29; 95% CI [.26, .33]) than in the neutral context (*M prop* = .36, 95% CI [.33, .39]; *M diff* = .06, 95% CI [.02, .11], *p* = .007; *d* = .92, 95% CI [.19, 1.67], but there was no difference between the masculine context and the feminine context (*M prop* = .32, 95% CI [.29, .35]; *M diff* = .03, 95% CI [−.02, .07], *p* = .236; *d* = .49, 95% CI [−.22, 1.19]), or between the feminine context and the neutral context (*M diff* = .04, 95% CI [−.01, .08], *p* = .115; *d* = .54, 95% CI [−.17, 1.25]).

*Is there greater evidence of stereotype maintenance for male targets than female targets?* Supporting the possibility that there was greater stereotype maintenance for male targets there was evidence of a Target Sex × Attribute Type interaction, *F*(1, 45) = 6.29, *p* = .016; η^2^ = .123. Stereotype-consistent attributes were paired more frequently with male targets (*M prop* = .40; 95% CI [.37, .43]) than female targets (*M prop* = .35; 95% CI [.33, .37]; *M diff* = .05; 95% CI [.01, .09], *p* = .010; *d* = .39, 95% CI [.09, .68]). Conversely, stereotype-inconsistent attributes were paired more frequently with female targets (*M prop* = .34; 95% CI [.32, .37]) than male targets (*M prop* = .31; 95% CI [.28, .33]; *M diff* = .04; 95% CI [.001, .07]; *p* = .044; *d* = .30, 95% CI [.01, .59]).

**Figure 6.**
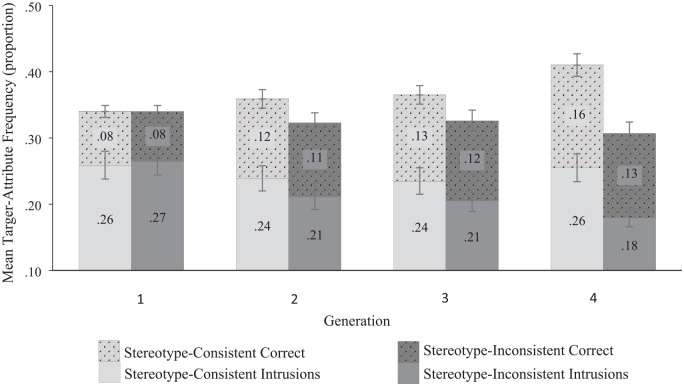
Experiments 1 to 3 Frequency of Target-Attribute Pairings by Generation, Attribute Type, and Accuracy. *Note.* Numbers in each bar represent the proportion of responses for each level and error bars denote 95% confidence intervals for each level.

*Are the effects driven by better memory for stereotype-consistent information or a bias toward making stereotype-consistent memory errors?* There was evidence of a significant Generation × Accuracy × Attribute Type interaction, *F*(1, 45) = 5.84, *p* = .020; η^2^ = .115; see Figure 6. Examination of the proportion of correct responses indicates that a memory advantage for stereotype-consistent correct responses emerged over time. At G1 there was no difference in the proportion of correct responses that were stereotype-consistent (*M prop* = .08; 95% CI [.07, .09]) relative to stereotype-inconsistent (*M prop* = .08; 95% CI [.07, .08]; *M diff* = .006; 95% CI [-.004, .017], *p* < .223; *d* = .18, 95% CI [−.11, .46]); however, by G4 there were significantly more correct responses that were stereotype-consistent (*M prop* = .16; 95% CI [.14, .17]) than stereotype-inconsistent (*M prop* = .13; 95% CI [.11, .15]; *M diff* = .03; 95% CI [.003, .05], *p* < .032; *d* = .31, 95% CI [.02, .60]). Although between G1 and G4 there was a significant increase in both stereotype-consistent correct responses (*M diff* = .07; 95% CI [.06, .09], *p* < .001; *d* = 1.21, 95% CI [.84, 1.59]) and stereotype-inconsistent correct responses (*M diff* = .05; 95% CI [.03, .07], *p* < .001; *d* = .76, 95% CI [.44, 1.08]), this increase was larger for stereotype-consistent correct responses.

Examination of the proportion of intrusions indicates that a bias toward stereotype-consistent intrusions emerged over time. At G1 there was no difference in the proportion of intrusions that were stereotype-consistent (*M prop* = .26; 95% CI [.24, .28]) relative to stereotype-inconsistent (*M prop* = .27; 95% CI [.24, .29]; *M diff* = .006; 95% CI [−.03, .04], *p* < .743; *d* = .05, 95% CI [−.24, 1.33]); however, by G4 there were significantly more intrusions that were stereotype-consistent (*M prop* = .26; 95% CI [.23, .28]) than stereotype-inconsistent (*M prop* = .18; 95% CI [.17, .20]; *M diff* = .08; 95% CI [.05, .10], *p* < .001; *d* = .75, 95% CI [.43, 1.07]). The emergent bias toward stereotype-consistent intrusions occurred because the proportion of stereotype-consistent intrusions did not change between G1 and G4 (*M diff* = .004; 95% CI [−.02, .03], *p* = .749; *d* = .04, 95% CI [−.24, .33]), whereas there was a significant decrease in stereotype-inconsistent intrusions between G1 and G4 (*M diff* = .08; 95% CI [−.02, .03], *p* < .001; *d* = 1.00, 95% CI [.65, 1.34]).

When the proportion of correct responses and intrusions are combined the effects further support those found in Experiments 1 to 3. At G1 there was no difference in the frequency of target-attribute pairings that were stereotype-consistent (*M prop* = .34; 95% CI [.32, .36]) relative to stereotype-inconsistent (*M prop* = .34; 95% CI [.32, .36]; *M diff* = .00; 95% CI [−.04, .40], *p* = .991; *d* = .002, 95% CI [−.28, .28]). As predicted, at G4 there was a significantly higher frequency of target-attribute pairings that were stereotype-consistent (*M prop* = .41; 95% CI [.38, .44]) than stereotype-inconsistent (*M prop* = .31; 95% CI [.29, .33]; *M diff* = .10; 95% CI [.06, .14]; *p* < .001; *d* = .66, 95% CI [.35, .97]). The pairwise comparisons also revealed that there were significantly more intrusions than correct responses for each combination of Attribute Type and Generation (all *p*s < .001).

*Is the size of stereotype-consistent memory bias the same across the generations or does it change as the social environment becomes increasingly stereotype-consistent?* We addressed the final aim of the cross-experiment analysis by investigating the level of stereotype-consistent bias added by each generation; we did this by subtracting the proportion of stereotype-consistent attribute pairings from the training phase from the proportion of stereotype-consistent attributes pairings from the test phase for each Generation. We then analyzed the stereotype-consistent memory bias within each Generation using a 3(Experiment Context: neutral vs. feminine vs. masculine) × 2(Generation: G1 vs. G4) × 2(Target Sex: female targets vs. male targets) × 2(Attribute Type: stereotype-consistent vs. stereotype-inconsistent) mixed ANOVA with Experiment Context as the only between-subjects factor (see Figure 6). The analysis revealed a main effect of Target Sex, *F*(1, 45) = 4.21, *p* = .046; η^2^ = .086, with a higher stereotype-consistent bias for male targets (*M prop* = .014; 95% CI [.006, .022]) than female targets (*M prop* = .014; 95% CI [−.001, .013]). There was also a significant main effect of Attribute Type, *F*(1, 45) = 6.15, *p* = .017; η^2^ = .120, but this was subsumed by a significant interaction of Generation × Attribute Type, *F*(1, 45) = 4.14, *p* = .048; η^2^ = .084, which is explored below. There was no evidence of any other significant main effects or interactions (all *F*s < 2.31, all *p*s > .111).

**Figure 7.**
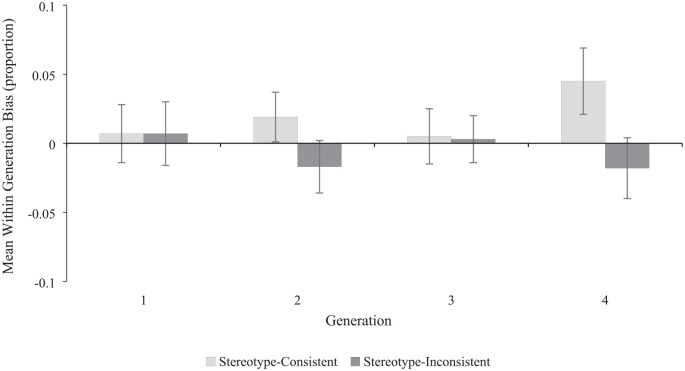
Expt. 1–3 Within Generation Bias by Experiment and Attribute Type. *Note*. Bias scores were calculated by subtracting the proportion of stereotype-consistent attribute pairings from the training phase from the proportion of stereotype-consistent attributes pairings from the test phase. Error bars represent 95% confidence intervals.

The Generation × Attribute Type interaction can be seen in Figure 7. Inspection of the pairwise comparisons revealed a significant increase in the frequency of stereotype-consistent bias between G1 (*M prop* = .007; 95% CI [−.014, .028]) and G4 (*M prop* = .045, 95% CI [.021, .069]; *M diff* = .038, 95% CI [.006, .070], *p* = .021; *d* = .35, 95% CI [.052, .634]), but no change in the frequency of stereotype-inconsistent bias between G1 (*M prop* = .007; 95% CI [−.016, .029]) and G4 (*M prop* = −.18, 95% CI [−.003, .040]; *M diff* = .025, 95% CI [−.011, .060], *p* = .167; *d* = .21, 95% CI [−.081, .491]). There was no difference between stereotype-consistent bias and stereotype-inconsistent bias at G1 (*M diff* < .001, 95% CI [−.40, .040], *p* = .991; *d* = .002, 95% CI [−.28, .28]), whereas there was a significant difference between stereotype-consistent bias and stereotype-inconsistent bias at G4 (*M diff* = .063, 95% CI [.022, .104], *p* = .003; *d* = .45, 95% CI [.15, .75]).

## Discussion—Cross-Experiment Analysis (Expt. 1-3)

The aim of the cross-experiment analysis was to establish whether the extent of stereotype maintenance differs depending on the target gender and social context and to further explore the mechanism underlying the effects. Supporting the trends from visual inspection of the means from Experiments 1 to 3, the cross-experiment analysis indicates there was stronger stereotype maintenance in the masculine context than in the neutral context and stronger stereotype maintenance for male targets than female targets. As information passed down the chains it became increasingly learnable, with significant increases in the proportion of stereotype-consistent and stereotype-inconsistent correct responses. Although there was no evidence of a memory bias toward stereotype-consistent information at the start of the chains—in either correct recall or memory intrusions—a memory bias for stereotype-consistent information emerged by the end of the chains. There was evidence that the strength of the stereotype-consistent memory bias changed across the generations, with a larger bias toward stereotype-consistent target-attribute pairings in the final generation than in the first generation.

The cross-experiment analysis suggests social information evolves to become increasingly gender stereotype-consistent over time and this process is mediated by both the target gender and a gendered social context (i.e., gendered occupations). Our findings are consistent with recent evidence that gender schemas are relative social constructs, which vary in their characteristics depending on the social context in which they are encountered (Martin, 2023). For example, Martin found that gender-neutral personality attributes were perceived to be more feminine when they appeared alongside masculine attributes but were perceived to be more masculine when they appeared alongside feminine attributes. Similarly, gender-neutral job candidates were perceived to be less masculine when they were preceded by a masculine candidate but were perceived to be less feminine when they were preceded by a feminine candidate. Martin’s findings suggest that gender schemas are not objective and fixed but are instead subjective and malleable depending on the context in which they appear (Martin, 2023). This begs the question of how the gender diversity of a social context might influence stereotype maintenance; for example, would one expect gender stereotypes to spontaneously re-emerge in a context where all targets were women or where all targets were men? We explored the influence of social context on stereotype maintenance in Experiment 4.

## Experiment 4—Single Gender Target Context

The aim of Experiment 4 was to examine whether the spontaneous re-emergence of gender stereotypes is influenced by the gender diversity of the social context in which they appear (i.e., single-sex context vs. mixed-sex context). Recent evidence suggests the effects of gender schema are dependent on the context in which stimuli appear, with targets perceived to be more feminine/masculine when they appear in a context with other stimuli that are more masculine/feminine, respectively (Martin, 2023). There is also evidence that the influence of gender schema is diminished when faces appear in a single-gender context (e.g., only women’s faces) relative to when they appear in a mixed-gender context (e.g., both women’s and men’ faces; Macrae & Cloutier, 2009; Martin et al., 2024). If gender schema are not objective and fixed but are instead subjective and malleable dependent on the context in which they appear (Macrae & Cloutier, 2009; Martin, 2023; Martin et al., 2024), it seems likely that this would influence the re-emergence of stereotypes via cumulative cultural evolution.

In Experiment 4, we investigated whether the contextual relationship between social targets influenced the maintenance of gender stereotypes via cultural evolution. Specifically, we examined whether gender stereotypes would be less likely to re-emerge in an intragroup context when all targets were from a single gender category, relative to an intergroup context when targets are from two gender categories (as in the previous experiments). The intergroup context was identical to that used in Experiment 3; the intragroup context perceivers were asked to learn about 16 targets, all of whom were female *or* all of whom were male (as opposed to 8 females *and* 8 males in Experiments 1-3). We chose to use the masculine job context from Expt. 3, as this produced the strongest evidence of stereotype maintenance. If encountering social targets in an intragroup context diminishes the influence of categorical person perception and stereotypes (e.g., Macrae & Cloutier, 2009; Martin, 2023; Martin et al., 2024), we would expect there to be less evidence of gender stereotype maintenance in the single-sex condition than in the mixed-sex condition. Specifically, we predicted that by the end of the chains, there would be significantly more stereotype-consistent target-attribute pairings in the mixed-sex condition than in the single-sex condition and significantly more stereotype-inconsistent target-attribute pairings in the single-sex condition than in the mixed-sex condition.

## Experiment 4—Method

### Participants

The participants were 256 18- to 30-year-olds (128 female and 128 female),^7^ half of whom were students recruited from the University of Aberdeen, who were granted course credit in exchange for taking part in the experiment, and half of whom were recruited online via Prolific Academic (www.prolific.ac), who completed the experiment remotely via the online testing platform *Gorilla* (www.gorilla.sc; Anwyl-Irvine et al., 2018), and were compensated around UK£6/US$7 for their time.

### Materials

The materials used were similar to those of Experiment 3, with the following exceptions. In the single-sex target condition, rather than participants seeing 16 targets, half of whom were female and half of whom were male, participants within a chain saw 16 targets that were either all female or all male. To this end, we created two new sets of target face images; one set of all female targets comprising the previous eight female target faces plus an additional eight female target faces, and one set of all male targets comprising the previous eight male target faces plus an additional eight male target faces. As before, the additional target faces were taken from the Chicago Face Database (Ma et al., 2015) and were subject to the same selection criteria. The mixed-sex target condition was identical to Experiment 3 with one exception; to exclude the possibility that the effects from Experiments 1 to 3 were driven by very specific target faces, we replaced the target images from Experiments 1 to 3 with the additional face images used to create the single-sex target sets.

### Procedure

For participants tested in the lab, the procedure was the same as that of Experiment 3. For participants tested online, we had no control over the physical environment in which they were tested. Participants were only able to complete the experiment on a laptop or desktop PC. The size and proportion of the face images were identical to Experiment 3 when presented on a 28-inch monitor with 1,280 × 1,024 screen resolution; the Gorilla testing platform standardizes the size ratio of the images across different screen sizes by asking participants to calibrate their screen size using a credit card.

### Dependent Measures and Analyses

The dependent measures and analyses were identical to those used in Experiments 1 to 3, with the following exceptions; we added the between-subjects factor of Target Sex Diversity and removed the within-subjects factor of Target Sex (because the latter was a between-subjects variable in the single-sex target condition and a within-subjects variable in the mixed-sex target condition).

## Experiment 4—Results

We analyzed the data for the mean proportional frequency with which targets were paired with gender-stereotyped attributes at the start and end of the chains using a 2 (Target Sex Context: single-sex targets vs. mixed-sex targets) × 2(Generation: G1 vs. G4) × 2 (Attribute Type: stereotype-consistent vs. stereotype-inconsistent) mixed ANOVA with Target Sex Context as a between-subjects factor (see Supplementary Table 6 for all cell values and Supplementary Table 7 for cell values including Target Sex). The analysis revealed significant main effects of Generation, *F*(1, 62) = 8.15, *p* = .006; η^2^ = .116, and Attribute Type, *F*(1, 62) = 5.06, *p* = .028; η^2^ = .075, and significant interactions of Target Sex Context × Attribute Type [*F*(1, 62) = 7.93, *p* = .007; η^2^ = .113] and Generation × Attribute Type, *F*(1, 62) = 4.88, *p* = .031; η^2^ = .073. However, all these effects were subsumed by the predicted significant interaction of Target Sex Context × Generation × Attribute Type, *F*(1, 62) = 4.76, *p* = .033; η^2^ = .071, which is explored below. There was no evidence of any other significant main effects or interactions (all *F*s < 2.10, all *p*s > .152).

**Figure 8.**
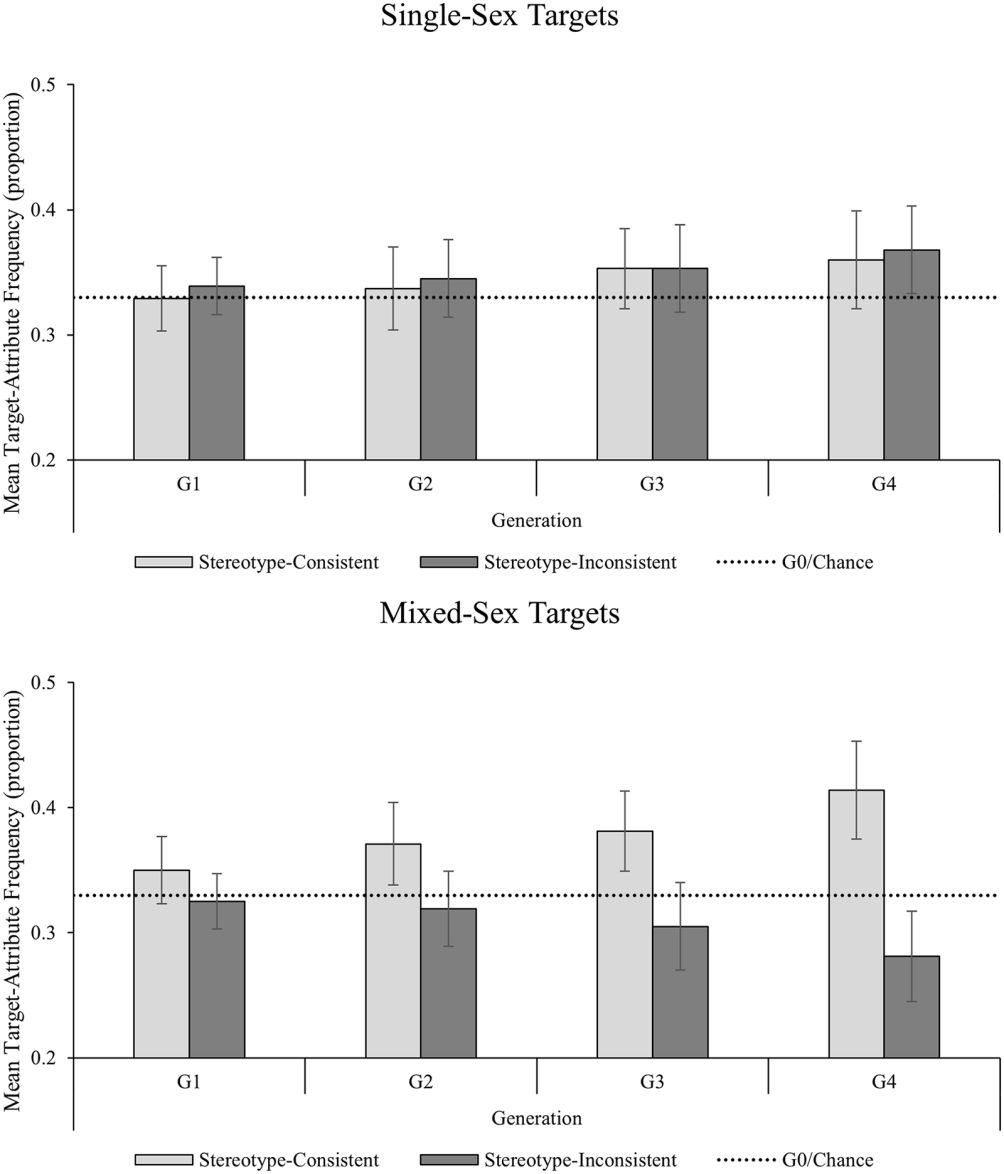
Expt. 4 Frequency of Target-Attribute Pairings by Generation and Attribute Type for the Single-Sex Context (Top Panel) and Mixed-Sex Context (Bottom Panel). *Note*. Error bars represent 95% confidence intervals.

We explored the Target Sex Context × Generation × Attribute Type interaction by examining the pairwise comparisons for stereotype-consistent and stereotype-inconsistent target-attribute pairings within and between Generation 1 and Generation 4 for the mixed-sex target and single-sex target conditions, respectively (Figure 8). The single-sex target condition diverged from Experiment 3, with no difference in stereotype-consistent target-attribute pairings between G1 (*M prop* = .33; 95% CI [.30, .36]) and G4 (*M prop* = .36; 95% CI [.32, .40]; *M diff* = .03 (95% CI [−.01, .07]; *p* = .163; *d* = .23, 95% CI [−.13, .58]; *r* = .163, *p* = .186), and no difference in stereotype inconsistent target-attribute pairings between G1 (*M prop* = .34; 95% CI [.32, .36]) and G4 (*M prop* = .37; 95% CI [.33, .40]; *M diff* = .03 (95% CI [−.007, .067]; *p* = .106; *d* = .27, 95% CI [−.08, .63]; *r* = .320, *p* = .037). While the mixed-sex target condition replicated the findings from Experiment 3, with a significant increase in stereotype-consistent target-attribute pairings between G1 (*M prop* = .35; 95% CI [.32, .38]) and G4 (*M prop* = .41; 95% CI [.38, .45]; *M diff* = .06 (95% CI [.02, .11]; *p* = .004; *d* = .59, 95% CI [.21, .96]; *r* = .173, *p* = .171), and a significant decrease in stereotype inconsistent target-attribute pairings between G1 (*M prop* = .33; 95% CI [.30, .35]) and G4 (*M prop* = .28; 95% CI [.25, .32]; *M diff* = .04 (95% CI [.008, .08]; *p* = .019; *d* = .46, 95% CI [.09, .82]; *r* = .264, *p* = .072). Consequently, by the end of the chains, there was no difference in the frequency of stereotype-consistent target-attribute pairings and stereotype-inconsistent target attribute pairings in the single-sex condition (*M diff* = .01 (95% CI [−.06, .08]; *p* = .799; *d* = .04, 95% CI [−.31, .39]; *r* = .632, *p* < .001), whereas there were significantly more stereotype-consistent target-attribute pairings than stereotype-inconsistent target attribute pairings in the mixed-sex condition (*M diff* = .13 (95% CI [.07, .20]; *p* < .001; *d* = .84, 95% CI [.43, 1.24]; *r* = −.458, *p* = .004).

## Discussion—Experiment 4

The aim of Experiment 4 was to examine whether the spontaneous re-emergence of gender stereotypes is influenced by the gender diversity of the social context in which they appear. The findings support and extend those from Experiments 1 to 3. As in Experiments 1 to 3, there was evidence information evolved as it passed along the chains, with significant changes in the frequency of stereotype-consistent and stereotype-inconsistent target-attribute pairings between the start and end of the chains. However, as predicted, this effect was modulated by the relative diversity of the social context with less evidence of gender stereotype maintenance in the single-sex condition than in the mixed-sex condition. Replicating the findings from Expt. 3, in a context containing both female and male targets gender stereotypes spontaneously re-emerged; however, in a single-sex context, gender stereotypes did not re-emerge. These findings support recent evidence that gender schema are relative social constructs, which are less likely to bias social cognition in single gender social contexts (e.g., Macrae & Cloutier, 2009; Martin, 2023; Martin et al., 2024).

## General Discussion

The aim of the current research was to establish whether societal stereotypes persist because stereotype-consistent memory bias incrementally shapes how social information evolves as it is repeatedly passed from person to person. Our findings support the possibility that, in the absence of communication bias, memory bias alone can cause social information to evolve to become increasingly stereotype-consistent. While we found some evidence of the re-emergence of gender stereotypes in Experiment 1, the effects were stronger in a feminine-stereotyped occupational context (Experiment 2) and a masculine-stereotyped occupational context (Expt. 3); conversely, the re-emergence of gender stereotypes was eliminated in a single gender context (Experiment 4). The current findings demonstrate that small amounts of cognitive bias, if widely shared, can cause existing gender stereotypes to be maintained through cultural evolution; however, they also suggest the maintenance of gender stereotypes is not the inevitable consequence of social transmission. Together, these findings have implications for our understanding of the memory biases that shape our individual experience and their cumulative consequences for the fabric of our society.

In line with gender schema theory (Bem, 1981; Liben & Bigler, 2017; Starr & Zurbriggen, 2017), the re-emergence of gender stereotypes was driven by a bias toward recalling stereotype-consistent information. Irrespective of whether one endorses stereotypes, schematic knowledge of stereotype content has the potential to influence the way one attends to, stores in memory, and recalls social information (Bem, 1981; Sebastián-Tirado et al., 2023). Often this results in a bias toward information that is consistent with one’s existing stereotype knowledge (Fyock & Stangor, 1994; Macrae & Bodenhausen, 2000; Rojahn & Pettigrew, 1992; Stangor & McMillan, 1992). In the current context, this bias toward gender stereotype-consistent information accumulated as information was repeatedly passed through the memories of the people in our chains. However, supporting the recent assertion that gender schema is a relative social construct (Martin, 2023), we also found that the extent to which gender stereotypes re-emerged was influenced by the social context in which targets appeared. Just as Martin found that perceptions of femininity/masculinity were dependent on the gendered context in which a stimulus appeared, so we found that the re-emergence of feminine/masculine stereotypes was dependent on the gendered context in which our targets appeared. Thus, at a macro-level, the likelihood that gender stereotypes re-emerge via cultural evolution is ultimately dependent at a micro-level on the likelihood that gender stereotypes bias the memories of individual people.

The re-emergence of gender stereotypes was driven by a bias toward recalling stereotype-consistent information irrespective of the accuracy of these recollections. The cross-experiment analysis revealed that although there was no evidence of a memory bias toward stereotype-consistent information at the start of the chains (in either correct recall or memory intrusions), a memory bias for stereotype-consistent information emerged by the end of the chains. Stereotypes re-emerged because people had a bias toward making stereotype-consistent intrusions (Bellezza & Bower, 1981; Lenton et al., 2001); indeed, it is unclear how the proportion of stereotype-consistent target-attribute pairings would have increased without a bias for stereotype-consistent intrusions. In reality, the nature of the current experiments is not well suited for determining whether people are better at accurately recalling stereotype-consistent information (Fyock & Stangor, 1994). This is because as the proportion of stereotype-consistent responses increases, so does the likelihood of higher accuracy for stereotype-consistent target-attribute pairings by chance. This issue is exacerbated as information passes down the chains, as a bias toward making more stereotype-consistent responses in the previous generation increases the proportion of stereotype-consistent target attribute pairings in the training phase of the current generation, which then further increases the likelihood of higher accuracy for stereotype-consistent target-attribute pairings by chance. Thus, the fact that the learning environment became increasingly stereotype-consistent might explain why people showed an increase in stereotype-consistent correct responses over time. However, irrespective of whether people are better at correctly recalling stereotype-consistent information, a bias toward stereotype-consistent memory intrusions can explain the re-emergence of stereotypes in the social environment.

The findings of the cross-experiment analysis suggest the strength of the stereotype-consistent memory bias increased as the social environment became increasingly stereotype-consistent. Participants at the end of the chains produced even more additional stereotype-consistent target attribute pairings than did participants at the start of the chains. As outlined above, in Generation 1, when the social environment did not contain gender stereotype-consistent bias, it appeared spontaneously through people’s small bias toward stereotype-consistent memory intrusions. However, by Generation 4, when the social environment did contain some gender stereotype-consistent bias, the size of people’s bias toward stereotype-consistent memory intrusions increased. Thus, the re-emergence of gender stereotypes at the end of the chains is not simply the accumulation of a uniform bias toward gender stereotype-consistent memory. It is an interactive process between the information in the environment and the way this information is cognitively represented. It is as though the stereotypes have been bootstrapped into re-emergence and that once they re-emerge, they exert an even greater influence on people’s cognitive representations and therefore the evolving environment.

While the cumulative effects of a small but widely shared bias toward gender stereotype-consistent memory might have negative consequences for society, the bias itself and the fact that it is widely shared might have adaptive consequences for individuals (Hutchison & Martin, 2015). People are only able to attend to, store, and accurately recall from memory a tiny proportion of the information that encounter in everyday life. This means there are many circumstances in which people are likely to suffer memory lapses when recalling information about other people. If these memory lapses were independent of gender stereotypes, then, as in our experiment, sometimes they would be accurate and sometimes they would not. However, in a world where women and men are more likely to identify themselves with gender stereotype-consistent attributes (Donnelly & Twenge, 2017), a bias toward gender stereotype-consistent intrusions increases the likelihood that one’s memory lapses will be accurate. In addition, if a bias toward gender stereotype-consistent memory intrusions is widely shared across the population, it increases the likelihood that people will share common ground with one another. Such stereotype-consistent common ground, even if based on error, can reduce uncertainty (Hogg & Reid, 2006), simplify communication (Stangor & Schaller, 2000), facilitate social interaction (Ruscher, 1998), and provide a foundation for a more structured and cohesive society (Bar-Tal, 2000).

Our findings suggest that even in the absence of social and communicative biases, a bias for stereotype-consistent memory causes information to evolve to become increasingly stereotype-consistent. Previous research suggests that people possess social and communicative biases that make them more likely to communicate stereotype-consistent information and that the effects of these biases accumulate over time (for a review see Kashima et al., 2007). It is possible that effects attributed to social or communicative bias might instead be driven by cognitive bias, as information must be recalled from memory before it can be communicated. However, in reality, people likely possess independent cognitive, social, and communicative stereotype-consistent biases for stereotype-consistent information all of which contribute to the re-emergence of existing stereotypes. By adapting the transmission method we use here, it would be relatively straightforward for future research to disambiguate the distinct and combined contributions of these different forms of bias to the re-emergence and maintenance of societal stereotypes (for analogous work in language evolution see Kirby et al., 2015; Ota et al., 2021).

The current findings highlight the importance of considering the cumulative effects of psychological bias, even when the effects of this bias are apparently very small at an individual level (Mesoudi, 2011). At Generation 1, there was no evidence of a significant memory bias toward stereotype-consistent information relative to stereotype-inconsistent information in any of the four experiments. Yet in Experiments 1 to 3, the modest levels of stereotype-consistent bias we see at each generation accumulated into much more substantial bias through repeated social transmission. In Experiment 4, the single gendered context appeared to reduce the level of stereotype-consistent bias (either within or across individuals), thereby prevented the spontaneous re-emergence of gender stereotypes. At the current time there is a substantial gap in our understanding of how social bias influences society. On the one hand, there is a large and growing experimental literature documenting lab-based social cognitive bias. On the contrary, there is abundant evidence of real-world social bias in people’s lived experience of individual and structural prejudice and discrimination. If many people share cognitive biases in the way they attend to, store, and recall social information (which we know they do), and if people repeatedly receive and transmit social information to others (which we know they do), then the likely consequence is directional cumulative cultural evolution (Hutchison & Martin, 2015; Martin et al., 2017). By examining the cumulative effects of social bias on culture, either through experimental work, through modeling or large-scale observational research, we might better understand how social cognitive bias continually shapes our society.

Interpretation and extrapolation of the findings from the present research are limited by the relative homogeneity of the social targets, workplace settings, and participant samples in our experiments. All targets in our experiments were White younger adults who worked in the an office environment; thus, gender was the only present primary category of social cognition (i.e., gender, race, and age; Fiske & Neuberg, 1990; Martin et al., 2015). Similarly, most participants were young, White women (although we did have equal numbers of men and women in Experiments 3 and 4). In reality, circumstances in which gender is the only relevant social category are rare. In typical real-world workplaces, for example, we might expect to see an array of ages, genders, and ethnicities, along with other, less primary categories such as job role, socio-economic status, religion, marital status, and sexual orientation. It would be worthwhile, then, to examine how stereotypes evolve when more than one such category is present—for example, in offices comprised of male and female, Black and White, younger and older adult co-workers, or among people who are employed in blue- and white-collar jobs, who are unemployed, or who are home caregivers (Sebastián-Tirado et al., 2023).

## Conclusion

Across four experiments, we aimed to establish whether societal gender stereotypes re-emerge and persist because stereotype-consistent memory bias shapes how social information evolves as it is repeatedly passed from person to person. The current findings demonstrate that, despite apparently changing societal attitudes and beliefs about gender, even small amounts of cognitive bias, if widely shared, might cause existing stereotype content to be maintained through cumulative cultural evolution. Thus, even if the external social environment were free from stereotypes, people’s gender schema mean that their cognitive representation of this environment would likely drive the spontaneous re-emergence of stereotypes as information is repeatedly socially transmitted. Yet, at the same time, our findings also suggest that this is not necessarily an inevitable consequence, and the way the social environment is structured might reduce the likelihood that stereotypes persist.

## Supplemental Material

sj-docx-1-psp-10.1177_01461672241254695 – Supplemental material for Many Mickles Make a Muckle: Evidence That Gender Stereotypes Reemerge Spontaneously Via Cultural Evolution Supplemental material, sj-docx-1-psp-10.1177_01461672241254695 for Many Mickles Make a Muckle: Evidence That Gender Stereotypes Reemerge Spontaneously Via Cultural Evolution by Carolyn J. Dallimore, Kenny Smith, Jacqui Hutchison, Gillian Slessor and Douglas Martin in Personality and Social Psychology Bulletin

## Notes

1. The term “cultural evolution” is broadly defined in the literature and has been applied to document how a diverse array of phenomena change over time, including but not limited to information, knowledge, behavior, attitudes, and beliefs (see Mesoudi, 2011).

2. Additional conditions: The current research was part of a wider project designed to separately examine how the cumulative cultural evolution of social information is influenced by both existing stereotype knowledge *and* intergroup bias associated with novel minimal groups. To this end, participants were told that the social targets they would be learning about belonged to the same fictitious team as themselves (i.e., an in-group) or a different team to themselves (i.e., an out-group). However, as was our original intention, in the current report we only examine the intended questions and data pertaining to the re-emergence of existing gender stereotypes. We examine questions and data pertaining to the influence of minimal intergroup bias in a separate report (this will be available from an open-source repository soon).

3. We collected femininity-masculinity ratings for each attribute (Likert-type scale 1 = most feminine – 5 = most masculine) using separate pilot samples of equivalent undergraduates (see Supplementary Table C). Subsequent analysis of these ratings revealed the mean ratings of feminine, masculine, and neutral items were all significantly different from one another (Supplementary Table D).

4. Because the data for the dependent measure are proportions of the whole, it is not appropriate to run an omnibus ANOVA with a factor of Attribute Type that has three levels (i.e., 3 (Attribute Type: stereotype-consistent vs. stereotype-inconsistent vs. stereotype neutral); doing so merely results in many comparisons between levels that have identical means and zero variance.

5. While our statistical analyses focus on the key difference in responses between the start and end of the chain (G1 vs. G4), we have graphically depicted the data for all four generations in the figures to illustrate the incremental nature of the cultural evolution process.

6. In Expt. 3, we recruited an equal number of female and male participants to minimize the possibility that the effects were being driven by participant sex or gender. Participants were tested in within participant gender chains (i.e., chains of four generations of women participants or chains of four generations of men participants). Preliminary exploratory analyses revealed there to be no significant effect of chain gender (although these analyses were underpowered).

7. As in Expt. 3, in Expt. 4 we recruited an equal number of female and male participants to minimize the possibility that the effects were being driven by participant sex or gender. Participants were tested in within participant gender chains; in half of the chains the targets were all female and in half the chains the targets were all male. While underpowered, preliminary exploratory analyses revealed no significant main effects or interactions involving Chain Sex (all *F*s < 3.44, all *p*s > .68).
