# Supplementary material for: Many Mickles Make a Muckle: Evidence That Gender Stereotypes Reemerge Spontaneously Via Cultural Evolution
Source: Pers Soc Psychol Bull. 2024 Jun 3;51(12):2405–24. doi: 10.1177/01461672241254695 (PMC12569133; doi:10.1177/01461672241254695)
Supplement: sj-docx-1-psp-10.1177_01461672241254695 – Supplemental material for Many Mickles Make a Muckle: Evidence That Gender Stereotypes Reemerge Spontaneously Via Cultural Evolution [file sj-docx-1-psp-10.1177_01461672241254695.docx]

Supplementary Table A. Target face image norms. Target image name refers to the original from the Chicago Face Database (Ma et al., 2015). Age is in years. All other measures are mean ratings on a 5-point Likert scale (1 = Least – 5 = Most)

| Expt. | Target Image Name | Target Sex | Feminine | Masculine | Attractive | Trustworthy | Unusual | Age |
| --- | --- | --- | --- | --- | --- | --- | --- | --- |
| 1 to 4 | WF-008 | F | 4.00 | 2.27 | 2.73 | 3.88 | 1.68 | 30.21 |
| 1 to 4 | WF-215 | F | 4.25 | 2.24 | 3.28 | 2.96 | 2.24 | 30.64 |
| 1 to 4 | WF-232 | F | 5.03 | 1.28 | 4.21 | 3.82 | 1.72 | 25.28 |
| 1 to 4 | WF-243 | F | 5.28 | 1.56 | 4.36 | 4.00 | 2.83 | 26.48 |
| 1 to 4 | WF-023 | F | 3.96 | 2.37 | 2.74 | 3.57 | 2.11 | 32.74 |
| 1 to 4 | WF-213 | F | 4.14 | 2.10 | 3.24 | 3.00 | 1.86 | 25.66 |
| 1 to 4 | WF-011 | F | 5.14 | 1.70 | 4.13 | 3.70 | 1.83 | 24.04 |
| 1 to 4 | WF-207 | F | 5.25 | 1.46 | 4.46 | 3.96 | 2.04 | 24.88 |
| 1 to 4 | WM-016 | M | 1.41 | 5.21 | 3.26 | 3.08 | 1.95 | 30.40 |
| 1 to 4 | WM-238 | M | 1.50 | 4.59 | 3.27 | 3.00 | 2.14 | 24.82 |
| 1 to 4 | WM-009 | M | 2.03 | 4.73 | 4.08 | 3.51 | 2.23 | 23.70 |
| 1 to 4 | WM-029 | M | 1.61 | 5.21 | 4.59 | 3.70 | 2.05 | 28.59 |
| 1 to 4 | WM-218 | M | 1.36 | 4.39 | 2.86 | 3.11 | 1.89 | 25.82 |
| 1 to 4 | WM-203 | M | 1.33 | 4.87 | 3.50 | 3.54 | 1.54 | 29.25 |
| 1 to 4 | WM-212 | M | 1.81 | 4.96 | 3.70 | 3.48 | 2.52 | 30.11 |
| 1 to 4 | WM-004 | M | 1.80 | 4.85 | 4.66 | 3.56 | 1.93 | 25.82 |
| 4 | WF-001 | F | 3.86 | 3.01 | 3.11 | 3.30 | 2.80 | 24.95 |
| 4 | WF-027 | F | 5.31 | 1.51 | 4.69 | 3.96 | 2.07 | 21.29 |
| 4 | WF-009 | F | 4.07 | 2.44 | 3.19 | 3.44 | 2.69 | 23.31 |
| 4 | WF-229 | F | 3.92 | 2.29 | 2.68 | 3.20 | 3.48 | 22.92 |
| 4 | WF-019 | F | 3.98 | 2.81 | 2.93 | 3.11 | 2.61 | 28.35 |
| 4 | WF-247 | F | 4.83 | 1.58 | 3.25 | 3.61 | 1.67 | 26.42 |
| 4 | WF-231 | F | 5.31 | 1.45 | 3.86 | 3.34 | 1.72 | 26.31 |
| 4 | WF-024 | F | 5.34 | 1.74 | 4.76 | 3.73 | 2.44 | 23.94 |
| 4 | WM-040 | M | 1.81 | 4.66 | 3.05 | 3.42 | 2.33 | 25.12 |
| 4 | WM-214 | M | 2.25 | 3.67 | 3.12 | 3.72 | 2.24 | 25.12 |
| 4 | WM-250 | M | 1.56 | 4.68 | 4.12 | 3.36 | 2.24 | 21.12 |
| 4 | WM-207 | M | 1.33 | 5.21 | 4.13 | 3.33 | 2.46 | 26.58 |
| 4 | WM-219 | M | 1.79 | 4.58 | 2.96 | 3.25 | 1.63 | 22.17 |
| 4 | WM-210 | M | 1.68 | 3.48 | 2.96 | 3.32 | 2.07 | 21.50 |
| 4 | WM-213 | M | 1.92 | 4.16 | 3.71 | 3.56 | 2.32 | 26.36 |
| 4 | WM-257 | M | 1.81 | 4.35 | 3.74 | 3.92 | 2.67 | 30.04 |

Supplementary Table B. Results of independent samples t-tests examining differences in mean ratings between Female and Male target faces. Age is in years; all other measures are Likert responses (1 = Most – 5 = Least).

|  | Female Targets | | Male Targets | | *t(30)* | *p* | Cohen's d |
| --- | --- | --- | --- | --- | --- | --- | --- |
|  | *M* | *SD* | *M* | *SD* |  |  |  |
| Femininity | 4.6 | 0.6 | 1.6 | 0.2 | 17.54 | < .001 | 6.20 |
| Masculinity | 1.9 | 0.4 | 4.9 | 0.3 | 14.58 | < .001 | 5.15 |
| Attractiveness | 3.6 | 0.7 | 3.8 | 0.6 | .053 | .958 | .02 |
| Trustworthiness | 3.6 | 0.4 | 3.4 | 0.3 | .980 | .334 | .35 |
| Unusualness | 2 | 0.4 | 2 | 0.3 | .693 | .494 | .25 |
| Age | 27.5 | 3.2 | 27.3 | 2.6 | .041 | .968 | .01 |

Supplementary Table C. Mean gender stereotype ratings for attributes from pilot testing (1 = feminine – 5 = masculine). During pilot testing, we asked a sample of 151 undergraduate students, who did not take part in the Experiments proper, to rate the relative gender stereotype of each attribute. The instructions given to participants can be seen overleaf.

| Attribute | *N* | *M* | *SD* | Attribute Type |
| --- | --- | --- | --- | --- |
| caring | 151 | 1.97 | .82 | Feminine |
| sympathetic | 151 | 2.06 | .76 | Feminine |
| compassionate | 150 | 2.06 | .77 | Feminine |
| affectionate | 150 | 2.09 | .86 | Feminine |
| soft spoken | 151 | 2.12 | .85 | Feminine |
| gentle | 151 | 2.20 | .86 | Feminine |
| innocent | 151 | 2.25 | .81 | Feminine |
| warm | 151 | 2.26 | 1.00 | Feminine |
| kind | 150 | 2.31 | .85 | Feminine |
| understanding | 151 | 2.35 | .76 | Feminine |
| encouraging | 151 | 2.40 | .80 | Feminine |
| gullible | 151 | 2.46 | .91 | Feminine |
| polite | 151 | 2.50 | .84 | Feminine |
| shy | 150 | 2.53 | .76 | Feminine |
| cheerful | 150 | 2.55 | .75 | Feminine |
| loyal | 151 | 2.56 | .68 | Feminine |
| jealous | 151 | 2.60 | .87 | Neutral |
| anxious | 151 | 2.61 | .76 | Neutral |
| tactful | 150 | 2.65 | .74 | Neutral |
| bitter | 150 | 2.68 | .82 | Neutral |
| committed | 151 | 2.74 | .78 | Neutral |
| friendly | 151 | 2.76 | .66 | Neutral |
| truthful | 150 | 2.85 | .73 | Neutral |
| secretive | 151 | 2.92 | .90 | Neutral |
| nasty | 151 | 2.97 | .93 | Neutral |
| lonely | 151 | 2.99 | .81 | Neutral |
| unpleasant | 151 | 3.07 | .99 | Neutral |
| selfish | 151 | 3.07 | .66 | Neutral |
| troublesome | 151 | 3.11 | .96 | Neutral |
| bullying | 151 | 3.13 | .67 | Neutral |
| thoughtless | 150 | 3.17 | .93 | Neutral |
| offensive | 151 | 3.23 | 1.03 | Neutral |
| original | 151 | 3.29 | .85 | Masculine |
| independent | 151 | 3.31 | .80 | Masculine |
| strong willed | 151 | 3.36 | .96 | Masculine |
| self-reliant | 151 | 3.40 | .97 | Masculine |
| strong personality | 151 | 3.40 | .78 | Masculine |
| analytical | 151 | 3.40 | .72 | Masculine |
| hostile | 151 | 3.45 | .84 | Masculine |
| self-sufficient | 149 | 3.50 | .84 | Masculine |
| confident | 151 | 3.56 | .87 | Masculine |
| ambitious | 150 | 3.57 | .84 | Masculine |
| assertive | 150 | 3.61 | .89 | Masculine |
| decisive | 151 | 3.70 | .98 | Masculine |
| competitive | 151 | 3.73 | .86 | Masculine |
| forceful | 151 | 3.92 | .80 | Masculine |
| dominant | 151 | 3.99 | .86 | Masculine |
| arrogant | 150 | 4.05 | .82 | Masculine |

Supplementary Table D. Results of paired samples t-tests examining differences in pilot test mean gender stereotype ratings for attributes grouped as feminine, masculine, and neutral (1 = feminine – 5 = masculine)

|  | *N* | *M* | *SD* | *t* | *p* | *d* |
| --- | --- | --- | --- | --- | --- | --- |
| Feminine vs. | 142 | 2.27 | .44 | 19.58 | < .001 | 1.64 |
| Masculine | 142 | 3.59 | .45 |  |  |  |
|  |  |  |  |  |  |  |
| Feminine vs. | 145 | 2.28 | .48 | 14.95 | < .001 | 1.24 |
| Neutral | 145 | 2.91 | .28 |  |  |  |
|  |  |  |  |  |  |  |
| Masculine vs. | 144 | 3.60 | .44 | 17.47 | < .001 | 1.46 |
| Neutral | 144 | 2.90 | .28 |  |  |  |

**Instructions for pilot ratings of attributes:**

In this survey we are interested in your knowledge of whether certain words are associated with the feminine and masculine stereotypes.

IMPORTANT - WE ARE NOT INTERESTED IN YOUR OWN PERSONAL BELIEFS

Please indicate how stereotypically feminine/masculine each of the words below are using the following scale:

1 = strongly associated with feminine stereotype

2 = more strongly associated with feminine than masculine stereotype

3 = not associated with either stereotype/equally associated with both stereotypes

4 = more strongly associated with masculine than feminine stereotype

5 = strongly associated with masculine stereotype

Supplementary Table E. Mean pilot ratings of how strongly associated personality attributes are with the fictitious Feminine and Masculine job titles. During pilot testing, we asked a sample of 180 undergraduate students (45 per job condition), who did not take part in the Experiments proper, to rate the relative gender stereotype of each attribute. The instructions given to participants can be seen overleaf.

| Feminine Stereotype Jobs | | |  | Masculine Stereotyped Jobs | | |
| --- | --- | --- | --- | --- | --- | --- |
| Attribute | *M* | *SD* |  | Attribute | *M* | *SD* |
| friendly (n) | 1.70 | 1.08 |  | analytical (m) | 1.99 | 1.23 |
| caring (f) | 1.74 | 1.08 |  | committed (n) | 2.15 | 1.17 |
| committed (n) | 1.75 | 1.07 |  | ambitious (m) | 2.16 | 1.08 |
| encouraging (f) | 1.78 | 1.23 |  | confident (m) | 2.19 | 1.07 |
| polite (f) | 1.81 | 1.03 |  | self-reliant (m) | 2.34 | 1.15 |
| understanding (f) | 1.81 | 1.10 |  | selfish (n) | 2.35 | 1.17 |
| kind (f) | 1.82 | 1.04 |  | assertive (m) | 2.36 | 1.01 |
| compassionate (f) | 1.86 | 1.06 |  | independent (m) | 2.39 | 1.17 |
| sympathetic (f) | 1.86 | 1.10 |  | decisive (m) | 2.41 | 1.19 |
| warm (f) | 1.94 | 1.08 |  | strong personality (m) | 2.50 | .99 |
| confident (m) | 2.03 | 1.12 |  | competitive (m) | 2.53 | 1.07 |
| loyal (f) | 2.05 | .99 |  | strong-willed (m) | 2.53 | 1.08 |
| truthful (n) | 2.14 | 1.14 |  | truthful (n) | 2.55 | 1.12 |
| affectionate (f) | 2.17 | .94 |  | encouraging (f) | 2.58 | .98 |
| cheerful (f) | 2.17 | 1.07 |  | original (m) | 2.59 | 1.06 |
| gentle (f) | 2.18 | .93 |  | tactful (n) | 2.64 | 1.09 |
| tactful (n) | 2.29 | 1.24 |  | loyal (f) | 2.66 | 1.10 |
| ambitious (m) | 2.34 | 1.01 |  | understanding (f) | 2.70 | 1.14 |
| decisive (m) | 2.44 | 1.11 |  | friendly (n) | 2.71 | 1.02 |
| self-reliant (m) | 2.45 | 1.13 |  | polite (f) | 2.79 | 1.02 |
| self-sufficient (m) | 2.47 | 1.08 |  | dominant (m) | 2.81 | 1.14 |
| original (m) | 2.61 | .89 |  | caring (f) | 2.89 | 1.14 |
| analytical (m) | 2.62 | 1.11 |  | cheerful (f) | 2.93 | .96 |
| strong-willed (m) | 2.65 | 1.10 |  | kind (f) | 2.98 | .97 |
| independent (m) | 2.68 | 1.17 |  | sympathetic (f) | 2.98 | 1.07 |
| strong personality (m) | 2.71 | 1.06 |  | warm (f) | 3.03 | .98 |
| assertive (m) | 2.78 | 1.07 |  | compassionate (f) | 3.06 | 1.01 |
| soft-spoken (f) | 3.01 | 1.24 |  | forceful (m) | 3.06 | 1.17 |
| innocent (f) | 3.06 | 1.02 |  | secretive (n) | 3.18 | 1.17 |
| competitive (m) | 3.38 | 1.16 |  | affectionate (f) | 3.23 | .97 |
| dominant (m) | 3.40 | 1.00 |  | gentle (f) | 3.23 | .94 |
| forceful (m) | 3.84 | 1.08 |  | innocent (f) | 3.33 | .98 |
| gullible (f) | 3.95 | 1.11 |  | arrogant (m) | 3.39 | 1.23 |
| shy (f) | 4.00 | 1.11 |  | self-sufficient (m) | 3.39 | 1.22 |
| secretive (n) | 4.01 | 1.16 |  | soft-spoken (f) | 3.58 | 1.05 |
| anxious (n) | 4.09 | 1.04 |  | offensive (n) | 3.60 | 1.37 |
| arrogant (m) | 4.09 | 1.14 |  | anxious (n) | 3.65 | 1.09 |
| lonely (n) | 4.12 | 1.14 |  | shy (f) | 3.65 | 1.16 |
| unpleasant (n) | 4.16 | 1.21 |  | unpleasant (n) | 3.65 | 1.21 |
| jealous (n) | 4.19 | 1.10 |  | troublesome (n) | 3.66 | 1.36 |
| thoughtless (n) | 4.21 | 1.21 |  | hostile (m) | 3.68 | 1.28 |
| bitter (n) | 4.22 | 1.22 |  | lonely (n) | 3.68 | 1.14 |
| selfish (n) | 4.23 | 1.23 |  | bitter (n) | 3.73 | 1.23 |
| troublesome (n) | 4.23 | 1.16 |  | bullying (n) | 3.74 | 1.38 |
| hostile (m) | 4.34 | 1.15 |  | nasty (n) | 3.76 | 1.29 |
| offensive (n) | 4.40 | 1.09 |  | jealous (n) | 3.80 | 1.21 |
| bullying (n) | 4.45 | 1.09 |  | thoughtless (n) | 3.91 | 1.29 |
| nasty (n) | 4.45 | 1.06 |  | gullible (f) | 4.00 | 1.23 |

Supplementary Table F. Results of paired samples t-tests examining whether each fictitious job title was more strongly associated with stereotypically feminine or masculine attributes (1 = most strongly associated – 5 = least strongly associated)

|  |  | Feminine  Attributes | | Masculine Attributes | |  |  |  |
| --- | --- | --- | --- | --- | --- | --- | --- | --- |
|  | *N* | *M* | *SD* | *M* | *SD* | *t* | *p* | Cohen's *d* |
| Mobility Analyst | 37 | 3.07 | .75 | 2.63 | .67 | 2.44 | .020 | .40 |
| Product Factors | 44 | 3.12 | .55 | 2.54 | .50 | 5.02 | < .001 | .77 |
| Social Resources | 38 | 2.44 | .69 | 2.92 | .58 | 3.21 | .001 | .52 |
| Community Outreach | 39 | 2.21 | .61 | 2.94 | .49 | 7.33 | < .001 | 1.17 |

**Example of instructions for pilot ratings of attributes associated with fictitious job titles:**

Which personality attributes do you associate with people who work in a "Mobility Analysts Team"?

In this survey we are interested in your opinion of how strongly associated personality attributes are with a member of a "Mobility Analysts Team" using the following scale:

1 = Definitely associated with "Mobility Analysts Team"

2 = Probably associated with "Mobility Analysts Team"

3 = Neutral

4 = Probably NOT associated with "Mobility Analysts Team"

5 = Definitely NOT associated with "Mobility Analysts Team"

Supplementary Table 1. Expt. 1 Mean proportion frequency of target-attribute pairings by Generation, Attribute Type, and Target Sex

|  |  | Female Targets | | |  | Male Targets | | |
| --- | --- | --- | --- | --- | --- | --- | --- | --- |
|  |  | Stereotype Consistent | Stereotype Inconsistent | Neutral |  | Stereotype Consistent | Stereotype Inconsistent | Neutral |
| Generation 1 | Mean | .280 | .379 | .341 |  | .333 | .337 | .329 |
|  | SD | .084 | .112 | .085 |  | .111 | .128 | .083 |
|  | N | 16 | 16 | 16 |  | 16 | 16 | 16 |
|  |  |  |  |  |  |  |  |  |
| Generation 2 | Mean | .319 | .361 | .320 |  | .387 | .314 | .299 |
|  | SD | .104 | .124 | .063 |  | .106 | .118 | .107 |
|  | N | 16 | 16 | 16 |  | 16 | 16 | 16 |
|  |  |  |  |  |  |  |  |  |
| Generation 3 | Mean | .306 | .401 | .293 |  | .374 | .340 | .286 |
|  | SD | .130 | .137 | .077 |  | .118 | .116 | .060 |
|  | N | 16 | 16 | 16 |  | 16 | 16 | 16 |
|  |  |  |  |  |  |  |  |  |
| Generation 4 | Mean | .358 | .376 | .266 |  | .411 | .335 | .254 |
|  | SD | .122 | .102 | .098 |  | .151 | .141 | .072 |
|  | N | 16 | 16 | 16 |  | 16 | 16 | 16 |

Supplementary Table 2. Expt. 2 Mean proportion frequency of target-attribute pairings by Generation, Attribute Type, and Target Sex

|  |  | Female Targets | | |  | Male Targets | | |
| --- | --- | --- | --- | --- | --- | --- | --- | --- |
|  |  | Stereotype Consistent | Stereotype Inconsistent | Neutral |  | Stereotype Consistent | Stereotype Inconsistent | Neutral |
| Generation 1 | Mean | .346 | .361 | .293 |  | .368 | .323 | .309 |
|  | SD | .064 | .094 | .068 |  | .108 | .106 | .074 |
|  | N | 16 | 16 | 16 |  | 16 | 16 | 16 |
|  |  |  |  |  |  |  |  |  |
| Generation 2 | Mean | .350 | .332 | .318 |  | .383 | .306 | .311 |
|  | SD | .130 | .091 | .123 |  | .124 | .131 | .090 |
|  | N | 16 | 16 | 16 |  | 16 | 16 | 16 |
|  |  |  |  |  |  |  |  |  |
| Generation 3 | Mean | .365 | .311 | .324 |  | .359 | .289 | .352 |
|  | SD | .132 | .113 | .097 |  | .097 | .096 | .101 |
|  | N | 16 | 16 | 16 |  | 16 | 16 | 16 |
|  |  |  |  |  |  |  |  |  |
| Generation 4 | Mean | .408 | .299 | .293 |  | .406 | .299 | .294 |
|  | SD | .139 | .118 | .099 |  | .117 | .093 | .110 |
|  | N | 16 | 16 | 16 |  | 16 | 16 | 16 |

Supplementary Table 3. Expt. 3 Mean proportion frequency of target-attribute pairings by Generation, Attribute Type, and Target Sex

|  |  | Female Targets | | |  | Male Targets | | |
| --- | --- | --- | --- | --- | --- | --- | --- | --- |
|  |  | Stereotype Consistent | Stereotype Inconsistent | Neutral |  | Stereotype Consistent | Stereotype Inconsistent | Neutral |
| Generation 1 | Mean | .335 | .319 | .346 |  | .378 | .320 | .302 |
|  | SD | .071 | .063 | .065 |  | .129 | .107 | .066 |
|  | N | 16 | 16 | 16 |  | 16 | 16 | 16 |
|  |  |  |  |  |  |  |  |  |
| Generation 2 | Mean | .335 | .353 | .312 |  | .383 | .272 | .345 |
|  | SD | .059 | .099 | .084 |  | .119 | .097 | .123 |
|  | N | 16 | 16 | 16 |  | 16 | 16 | 16 |
|  |  |  |  |  |  |  |  |  |
| Generation 3 | Mean | .336 | .363 | .301 |  | .448 | .250 | .302 |
|  | SD | .093 | .112 | .079 |  | .127 | .118 | .092 |
|  | N | 16 | 16 | 16 |  | 16 | 16 | 16 |
|  |  |  |  |  |  |  |  |  |
| Generation 4 | Mean | .375 | .314 | .311 |  | .499 | .221 | .280 |
|  | SD | .126 | .118 | .091 |  | .121 | .100 | .107 |
|  | N | 16 | 16 | 16 |  | 16 | 16 | 16 |

Supplementary Table 4. Expt. 1-3 Mean proportion frequency of target-attribute pairings by Generation, Attribute Type, and Target Sex

|  |  | Female Targets | | |  | Male Targets | | |
| --- | --- | --- | --- | --- | --- | --- | --- | --- |
|  |  | Stereotype Consistent | Stereotype Inconsistent | Neutral |  | Stereotype Consistent | Stereotype Inconsistent | Neutral |
| Generation 1 | Mean | .320 | .353 | .327 |  | .360 | .327 | .313 |
|  | SD | .078 | .094 | .076 |  | .115 | .112 | .074 |
|  | N | 48 | 48 | 48 |  | 48 | 48 | 48 |
|  |  |  |  |  |  |  |  |  |
| Generation 2 | Mean | .335 | .349 | .317 |  | .384 | .297 | .319 |
|  | SD | .101 | .104 | .092 |  | .114 | .115 | .107 |
|  | N | 48 | 48 | 48 |  | 48 | 48 | 48 |
|  |  |  |  |  |  |  |  |  |
| Generation 3 | Mean | .336 | .359 | .306 |  | .394 | .293 | .313 |
|  | SD | .120 | .124 | .084 |  | .119 | .114 | .089 |
|  | N | 48 | 48 | 48 |  | 48 | 48 | 48 |
|  |  |  |  |  |  |  |  |  |
| Generation 4 | Mean | .380 | .330 | .290 |  | .439 | .285 | .276 |
|  | SD | .128 | .115 | .096 |  | .135 | .121 | .097 |
|  | N | 48 | 48 | 48 |  | 48 | 48 | 48 |

Supplementary Table 5. Cross-experiment ANOVA results. 3(Experiment Context: neutral vs. feminine vs. masculine) X 2(Generation: G1 vs. G4) X 2(Target Sex: female targets vs. male targets) X 2(Attribute Type: stereotype-consistent vs. stereotype-inconsistent) X 2(Accuracy: correct vs. intrusions).

| Effect | Sum of Squares | *df* | *MSE* | *F* | *p* | η^2^ |
| --- | --- | --- | --- | --- | --- | --- |
| Intercept | 23.42 | 1 | 23.42 | 6107.00 | <.001 | .993 |
| Experiment | .002 | 2 | .001 | .22 | .805 | .010 |
| Error (Experiment) | .173 | 45 | .004 |  |  |  |
|  |  |  |  |  |  |  |
| Generation | .017 | 1 | .017 | 13.27 | <.001 | .228 |
| Generation * Experiment | .010 | 2 | .005 | 3.93 | .027 | .149 |
| Error (Generation) | .056 | 45 | .001 |  |  |  |
|  |  |  |  |  |  |  |
| Accuracy | 3.252 | 1 | 3.252 | 386.27 | <.001 | .896 |
| Accuracy * Experiment | .065 | 2 | .032 | 3.85 | .029 | .146 |
| Error (Accuracy) | .379 | 45 | .008 |  |  |  |
|  |  |  |  |  |  |  |
| Attribute Type | .125 | 1 | .125 | 9.45 | .004 | .174 |
| Attribute Type * Experiment | .106 | 2 | .053 | 4.01 | .025 | .151 |
| Error (Attribute Type) | .597 | 45 | .013 |  |  |  |
|  |  |  |  |  |  |  |
| Target Sex | .002 | 1 | .002 | 1.72 | .196 | .037 |
| Target Sex * Experiment | .004 | 2 | .002 | 1.65 | .204 | .068 |
| Error (Target Sex) | .059 | 45 | .001 |  |  |  |
|  |  |  |  |  |  |  |
| Generation * Accuracy | .546 | 1 | .546 | 88.87 | <.001 | .664 |
| Generation * Accuracy * Experiment | .066 | 2 | .033 | 5.38 | .008 | .193 |
| Error (Generation*Accuracy) | .277 | 45 | .006 |  |  |  |
|  |  |  |  |  |  |  |
| Generation * Attribute Type | .124 | 1 | .124 | 18.60 | <.001 | .292 |
| Generation * Attribute Type * Experiment | .006 | 2 | .003 | .45 | .640 | .020 |
| Error (Generation*Attribute Type) | .301 | 45 | .007 |  |  |  |
|  |  |  |  |  |  |  |
| Accuracy * Attribute Type | .015 | 1 | .015 | 2.25 | .140 | .048 |
| Accuracy * Attribute Type * Experiment | .021 | 2 | .010 | 1.60 | .213 | .067 |
| Error (Accuracy*Attribute Type) | .293 | 45 | .007 |  |  |  |
|  |  |  |  |  |  |  |
| Generation * Accuracy * Attribute Type | .043 | 1 | .043 | 5.84 | .020 | .115 |
| Generation * Accuracy * Attribute Type * Experiment | .014 | 2 | .007 | .93 | .401 | .040 |
| Error (Generation*Accuracy*Attribute Type) | .329 | 45 | .007 |  |  |  |
|  |  |  |  |  |  |  |
| Generation * Target Sex | .000 | 1 | .000 | .00 | .982 | .000 |
| Generation * Target Sex * Experiment | .000 | 2 | .000 | .17 | .842 | .008 |
| Error (Generation*Target Sex) | .049 | 45 | .001 |  |  |  |
|  |  |  |  |  |  |  |
| Accuracy * Target Sex | .031 | 1 | .031 | 12.36 | .001 | .215 |
| Accuracy * Target Sex * Experiment | .001 | 2 | .000 | .15 | .865 | .006 |
| Error (Accuracy*Target Sex) | .112 | 45 | .002 |  |  |  |
|  |  |  |  |  |  |  |
| Generation * Accuracy * Target Sex | .000 | 1 | .000 | .11 | .743 | .002 |
| Generation * Accuracy * Target Sex * Experiment | .000 | 2 | .000 | .08 | .925 | .003 |
| Error (Generation*Accuracy*Target Sex) | .123 | 45 | .003 |  |  |  |
|  |  |  |  |  |  |  |
| Attribute Type * Target Sex | .086 | 1 | .086 | 6.29 | .016 | .123 |
| Attribute Type * Target Sex * Experiment | .021 | 2 | .010 | .76 | .476 | .032 |
| Error (Attribute Type*Target Sex) | .612 | 45 | .014 |  |  |  |
|  |  |  |  |  |  |  |
| Generation * Attribute Type * Target Sex | .004 | 1 | .004 | .49 | .488 | .011 |
| Generation * Attribute Type * Target Sex * Experiment | .030 | 2 | .015 | 1.71 | .192 | .071 |
| Error (Generation*Attribute Type*Target Sex) | .394 | 45 | .009 |  |  |  |
|  |  |  |  |  |  |  |
| Accuracy * Attribute Type * Target Sex | .021 | 1 | .021 | 2.99 | .091 | .062 |
| Accuracy * Attribute Type * Target Sex * Experiment | .011 | 2 | .006 | .83 | .442 | .036 |
| Error (Accuracy*Attribute Type*Target Sex) | .310 | 45 | .007 |  |  |  |
|  |  |  |  |  |  |  |
| Generation * Accuracy * Attribute Type * Target Sex | .000 | 1 | .000 | .04 | .853 | .001 |
| Generation * Accuracy * Attribute Type * Target Sex * Experiment | .001 | 2 | .000 | .05 | .952 | .002 |
| Error (Generation*Accuracy*Attribute Type*Target Sex) | .263 | 45 | .006 |  |  |  |

Supplementary Table 6. Expt. 6 Mean proportion frequency of target-attribute pairings by Target Sex Context, Generation, Target Sex, and Attribute Type

|  |  | Single Sex Targets | | |  | Mixed Sex Targets | | |
| --- | --- | --- | --- | --- | --- | --- | --- | --- |
|  |  | Stereotype Consistent | Stereotype Inconsistent | Neutral |  | Stereotype Consistent | Stereotype Inconsistent | Neutral |
| Generation 1 | Mean | .329 | .339 | .333 |  | .350 | .326 | .320 |
|  | SD | .079 | .060 | .069 |  | .072 | .068 | .070 |
|  | N | 32 | 32 | 32 |  | 32 | 32 | 32 |
|  |  |  |  |  |  |  |  |  |
| Generation 2 | Mean | .337 | .345 | .319 |  | .371 | .319 | .304 |
|  | SD | .103 | .097 | .072 |  | .080 | .075 | .087 |
|  | N | 32 | 32 | 32 |  | 32 | 32 | 32 |
|  |  |  |  |  |  |  |  |  |
| Generation 3 | Mean | .353 | .353 | .293 |  | .381 | .305 | .309 |
|  | SD | .102 | .110 | .088 |  | .075 | .083 | .106 |
|  | N | 32 | 32 | 32 |  | 32 | 32 | 32 |
|  |  |  |  |  |  |  |  |  |
| Generation 4 | Mean | .360 | .369 | .272 |  | .414 | .281 | .301 |
|  | SD | .122 | .113 | .101 |  | .095 | .089 | .095 |
|  | N | 32 | 32 | 32 |  | 32 | 32 | 32 |

Supplementary Table 7. Expt. 4 Mean proportion frequency of target-attribute pairings by Target Sex Context, Target Sex, Generation, and Attribute Type

|  |  |  | Female Targets | | |  | Male Targets | | |
| --- | --- | --- | --- | --- | --- | --- | --- | --- | --- |
|  |  |  | Stereotype Consistent | Stereotype Inconsistent | Neutral |  | Stereotype Consistent | Stereotype Inconsistent | Neutral |
| Single Sex Target Context | Generation 1 | Mean | .320 | .350 | .330 |  | .339 | .328 | .334 |
|  |  | SD | .080 | .068 | .073 |  | .080 | .050 | .065 |
|  |  | N | 16 | 16 | 16 |  | 16 | 16 | 16 |
|  |  |  |  |  |  |  |  |  |  |
|  | Generation 2 | Mean | .316 | .352 | .331 |  | .357 | .337 | .306 |
|  |  | SD | .098 | .116 | .080 |  | .108 | .075 | .066 |
|  |  | N | 16 | 16 | 16 |  | 16 | 16 | 16 |
|  |  |  |  |  |  |  |  |  |  |
|  | Generation 3 | Mean | .305 | .398 | .297 |  | .402 | .307 | .291 |
|  |  | SD | .088 | .116 | .094 |  | .094 | .084 | .082 |
|  |  | N | 16 | 16 | 16 |  | 16 | 16 | 16 |
|  |  |  |  |  |  |  |  |  |  |
|  | Generation 4 | Mean | .313 | .423 | .264 |  | .407 | .315 | .279 |
|  |  | SD | .094 | .099 | .087 |  | .132 | .102 | .116 |
|  |  | N | 16 | 16 | 16 |  | 16 | 16 | 16 |
|  |  |  |  |  |  |  |  |  |  |
| Mixed Sex Target Context | Generation 1 | Mean | .323 | .340 | .337 |  | .376 | .312 | .313 |
|  |  | SD | .101 | .108 | .128 |  | .098 | .078 | .072 |
|  |  | N | 32 | 32 | 32 |  | 32 | 32 | 32 |
|  |  |  |  |  |  |  |  |  |  |
|  | Generation 2 | Mean | .329 | .343 | .328 |  | .413 | .296 | .292 |
|  |  | SD | .118 | .128 | .130 |  | .125 | .112 | .103 |
|  |  | N | 32 | 32 | 32 |  | 32 | 32 | 32 |
|  |  |  |  |  |  |  |  |  |  |
|  | Generation 3 | Mean | .331 | .347 | .322 |  | .430 | .262 | .308 |
|  |  | SD | .112 | .152 | .135 |  | .142 | .132 | .127 |
|  |  | N | 32 | 32 | 32 |  | 32 | 32 | 32 |
|  |  |  |  |  |  |  |  |  |  |
|  | Generation 4 | Mean | .350 | .331 | .319 |  | .477 | .231 | .293 |
|  |  | SD | .161 | .127 | .121 |  | .136 | .112 | .132 |
|  |  | N | 32 | 32 | 32 |  | 32 | 32 | 32 |
